# Tissue Perfusion and Biomarkers Assessment Following Root Coverage Procedures

**DOI:** 10.1111/jre.13374

**Published:** 2025-01-22

**Authors:** Lorenzo Tavelli, Tu Nguyen, Maria Vera Rodriguez, Leonardo Mancini, William V. Giannobile, Shayan Barootchi

**Affiliations:** ^1^ Division of Periodontology, Department of Oral Medicine, Infection, and Immunity Harvard School of Dental Medicine Boston Massachusetts USA; ^2^ Department of Periodontics & Oral Medicine University of Michigan School of Dentistry Ann Arbor Michigan USA; ^3^ School of Dentistry Universidad Catolica de Santiago de Guayaquil (UCSG) Guayaquil Ecuador; ^4^ Center for Clinical Research and Evidence Synthesis in Oral TissuE RegeneratION (CRITERION) Boston Massachusetts USA; ^5^ Postgraduate Periodontics, Division of Periodontics Columbia University College of Dental Medicine New York City New York USA; ^6^ Clinic of Reconstructive Dentistry, Centre of Dental Medicine University of Zurich Zurich Switzerland

**Keywords:** blood flow, clinical trial, collagen matrix, gingival recession, growth factor, ultrasonography

## Abstract

**Aim:**

To assess tissue perfusion changes and wound healing biomarker levels after root coverage procedures with coronally advanced flap in combination with the cross‐linked xenogeneic collagen matrix (CCMX), loaded either with a placebo or recombinant human platelet‐derived growth factor‐BB (rhPDGF).

**Methods:**

This study was designed as a secondary analysis from a previously published clinical trial, and it assessed the tissue perfusion changes over 6 months around multiple gingival recession defects, treated with either with CCMX alone (control) or with CCMX + rhPDGF (test). High frequency Doppler ultrasonography (HFUS) scans were obtained at sites of interest at baseline, 2 weeks, 3 months, and 6 months after surgery. Dynamic tissue perfusion measurements (DTPMs) were performed at the midfacial, interproximal, and transverse aspects of the teeth by an operator, blinded to treatment allocation, using a software package. The expression of different wound healing biomarkers from the gingival crevicular fluid was also assessed.

**Results:**

The regression analyses showed similar tissue perfusion changes between the two groups throughout the majority of the 6 months. DTPMs at 2 weeks showed the test group to have significantly higher perfusion relief intensity (pRI, *p* < 0.001), mean perfused area (pA, *p* < 0.001), mean blood flow intensity (FI_mean_, *p* = 0.021), and total blood flow intensity (FI_tot_, *p* = 0.021) at the graft region of interest (ROI) compared to control sites. The test sites also exhibited significantly greater pA (*p* = 0.033) and blood flow intensity “blue” (FI_blue_, meaning flow away from the transducer, *p* = 0.035) at the level of the flap compared to the control sites. At 2 weeks, FI_blue_ of the graft was directly correlated with the final mean root coverage (*p* = 0.008) and complete root coverage (*p* = 0.003). FI_mean_ and FI_tot_ of the graft exhibited a direct correlation with volume gain at 6 months (*p* = 0.031 for both parameters). The final GT gain was correlated to the early DTPMs (pA and FI_blue_) of the graft and the flap. The two groups exhibited different expressions of IL‐1β, PDFG‐BB, and VEGF over 3 months, with the 1‐week levels of PDGF‐BB that were associated with time to recovery.

**Conclusions:**

HFUS allowed exquisite assessment of tissue perfusion occurring at the entire surgical reconstructive regions and also within the flap and the graft. Sites treated with CCMX + rhPDGF exhibited higher DTPMs, primarily within the graft and flap ROIs at the 2‐week timepoint compared to sites augmented with CCMX + saline. Early DTPMs at the graft and flap ROIs showed associations with PROMs and the final clinical outcomes.

**Trial Registration:** ClinicalTrials.gov: NCT04462237

## Graphical abstract

Gingival recessions treated with a collagen matrix loaded with rhPDGF showed significantly greater early tissue perfusion compared to sites treated without the growth factor. Early tissue perfusion outcomes were correlated to the amount of root coverage and gingival thickness gain at 6 months.

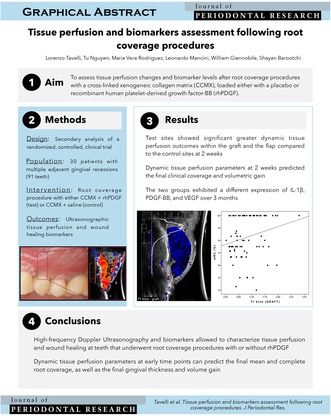

**Summary**

- Background
  ○ Doppler high frequency ultrasonography (HFUS) has been extensively used in the medical field, while its application around teeth is much less explored.
- Added value of this study
  ○ The present study depicted changes in ultrasonographic tissue perfusion and in the levels of wound healing biomarkers following the root coverage procedure using a xenogeneic collagen matrix (CCMX), either in combination with recombinant human platelet‐derived growth factor‐BB (rhPDGF), or an identical placebo agent.
  ○ HFUS allowed discrimination of tissue perfusion dynamics at the entire soft tissue area and also at the level of the graft and the flap regions at 2‐week post‐surgery.
- Clinical implications
  ○ Loading CCMXs with rhPDGF resulted in significantly higher tissue perfusion outcomes within the matrix and the flap at 2 weeks, which may explain the superior clinical and volumetric outcome obtained with CCMX combined with rhPDGF.
  ○ Early tissue perfusion parameters may predict the final clinical outcomes of soft tissue grafting for root coverage procedures.

## 1 Introduction

Gingival recession is a common condition affecting a large proportion of the population [1, 2, 3]. Soft tissue graft‐based root coverage procedures have been proved effective in the treatment of gingival recessions [4, 5, 6]. Among the commonly employed techniques, on average, the autogenous connective tissue graft is the approach associated with the highest coverage of recession defects [4, 7]. Nevertheless, there are also drawbacks related to the use of autogenous connective tissue graft, including an additional surgical area, increased patient post‐operative morbidity, risk of injure the greater palatine artery, and profuse intra‐ and/or post‐operative bleeding from the donor site, among others [8, 9]. These limitations are more pronounced when it comes to multiple adjacent gingival recessions (MAGRs) [10, 11]. Therefore, in order to promote a more patient‐centered and minimally invasive treatment of gingival recessions, several soft tissue graft substitutes have been investigated for root coverage purposes [10, 11, 12]. It has also been advocated that their combination with biologic agents could further enhance the root coverage outcomes [13]. In vitro studies demonstrated that loading a novel cross‐linked xenogeneic collagen matrix (CCMX) with recombinant human platelet‐derived growth factor‐BB (rhPDGF) promoted an increased cellular population and metabolic activity within the matrix [14] and further boosted the properties of the CCMX of stabilizing the blood clot and regulating the equilibrium between coagulation and fibrinolysis [15]. rhPDGF is known for enhancing angiogenesis and promoting the recruitment and activation of fibroblasts, accelerating the rate of wound healing [16, 17, 18]. This growth factor has been used in oral regenerative procedures for over two decades [13, 19]. Our group performed a randomized, controlled, and clinical trial investigating the effect of rhPDGF in combination with CCMX for the treatment of MAGRs. At 6 months, superior root coverage outcomes, in terms of higher mean root coverage (mRC), volumetric gain, gingival thickness (GT) gain, and professional esthetic scores, were obtained at sites treated with CCMX loaded with rhPDGF, compared to sites that received a saline‐saturated (as placebo) CCMX. While it could be speculated that these findings could be due to the mechanism of action of rhPDGF, that may have accelerated the process of vascularization of the CCMX and the resolution of the inflammatory phase [18, 20, 21], advances in imaging technology may allow to investigate, in a non‐invasive manner, the early wound healing phenomena occurring at teeth grafted with a CCMX, either with or without rhPDGF.

The use of high frequency Doppler ultrasonography (HFUS) has recently gained popularity in the dental setting [22, 23, 24]. This imaging tool allows to assess—in a non‐invasive manner—the components of the periodontal and peri‐implant phenotype, including the thickness of the soft and hard tissue [25, 26, 27, 28]. Recent studies have highlighted the benefits of combining HFUS to the conventional diagnostic methods to discriminate periodontal and peri‐implant health vs. disease [22, 23]. Furthermore, HFUS is able to visualize blood flow by employing the acoustic signal phase‐shift effects, based on the relative frequency shifts of the received echoes as a result of the movement of red blood cells in the vessels [24, 29]. This modality has been extensively utilized in the medical field for visualizing and quantifying the blood flow within grafts, lesions, and organs [30, 31, 32, 33]. In the dental field, ultrasonographic tissue perfusion assessment has been investigated for discriminating blood flow at healthy and diseased implant sites [34] and for evaluating the revascularization of connective tissue grafts after peri‐implant soft tissue augmentation [35, 36]. It is reasonable to assume that HFUS may be able to provide additional insights into the wound healing events occurring in natural dentition after root coverage procedure with a CCMX, either alone or saturated with rhPDGF.

Therefore, the aim of the present study was to utilize HFUS to characterize and quantify tissue perfusion at teeth with MAGRs that had been treated with CCMX + placebo and CCMX + rhPDGF, to explore the significance of early blood flow‐related parameters on the final root coverage outcomes. The expression of oral wound healing biomarkers from the gingival crevicular fluid (GCF) within the two groups was also assessed.

## 2 Methods

### 2.1 Study Design and Trial Registration

The present study was designed as a secondary analysis of a previously published triple‐blind, parallel‐arm, randomized, placebo‐controlled clinical trial [18], assessing the efficacy of rhPDGF in combination with a xenogeneic cross‐linked collagen matrix (CCMX) (CCMX + rhPDGF, test group) versus CCMX + sterile saline (CCMX + placebo, control group) for the treatment of multiple adjacent gingival recessions (MAGRs). The clinical and volumetric outcomes have been previously reported [18]. The clinical trial was registered prior to initiation at ClinicalTrials.gov and follows the CONSORT statement [37, 38] (Figure S1). The protocol of the study was approved by the Institutional Review Board of the University of Michigan Medical School (HUM00177214), in accordance with the Declaration of Helsinki of 1975, revised in Fortaleza in 2013. Informed consents were obtained from all participating individuals in this research. The present manuscript aimed at exploring ultrasonographic tissue perfusion and biomarkers levels at teeth treated with either CCMX + saline or CCMX + rhPDGF.

### 2.2 Participants

Thirty subjects presenting with the following inclusion and exclusion criteria were enrolled and treated. Inclusion criteria are as follows: (i) periodontally and systemically healthy adults (age ≥ 18 years) presenting with at least 2 MAGRs classified as recession type 1 (RT1) [39], associated with dental hypersensitivity or esthetic concerns; (ii) self‐reported smoking ≤ 10 cigarettes/day; (iii) full‐mouth plaque and bleeding scores ≤ 20%; (iv) presence of a least 2 mm depth on at least one recession; and (v) patients being able to maintain good oral hygiene. The exclusion criteria included the following: (i) compromised general health, (ii) pregnancy or attempting to get pregnant (self‐reported), (iii) untreated periodontal disease, (iv) persistence of uncorrected factitious gingival trauma from toothbrushing, (v) presence of severe tooth malposition, rotation, or super‐eruption, (vi) presence of root caries or inadequate prosthetic restorations, (vii) previous periodontal plastic surgery at the experimental sites, and (viii) known allergy to collagen‐based medical products.

### 2.3 Intervention

The surgeon, the patients, and the other study team members were blinded and remained uninformed of the treatment allocation (test or control group). On the day of the surgery, the surgeon received a sealed envelope with the patient's ID number, containing a syringe with a 1.5 cc of a clear solution (either sterile saline or rhPDGF). A detailed description of the intervention is depicted in Appendix S1 and in the original publication [18]. Briefly, the intervention consisted of coronally advanced flap (CAF) with a CCMX (Geistlich Fibro‐Gide, Geistlich Pharma AG, Wolhusen, Switzerland), either saturated with a sterile saline solution (control group) or with rhPDGF (GEM 21S, Lynch Biologics, Franklin, TN, USA, test group). Based on the location and distribution of the MAGRs, CAF was performed with a trapezoidal or envelope design, with horizontal or rotated papillae, with or without vertical incisions, as previously described [40, 41, 42]. After trimming extraorally the CCMX (Table S1), the graft was saturated with micro‐injections of a 1.5 cc solution (either saline or rhPDGF) that was prepared and provided by another study member through a sealed envelope. The CCMXs were left in the dappen dish for 15 min, with the solution that was also applied onto the dried root surfaces before suturing the graft. Simple interrupted sutures (6/0 and 7/0 PGA, AD Surgical, Sunnyvale, USA) engaging the CCMX and the de‐epithelialized anatomical papillae were performed for stabilizing the graft at the recipient bed, approximately at the level of the cemento‐enamel junction (CEJ) or 1 mm apical. Further stabilization of the matrix was also achieved, if necessary, with additional mattress sutures apical to the CCMX, through engaging the periosteum. The flap, that was previously released, was then coronally advanced and stabilized approximately 2 mm above the CEJ using sling sutures and simple interrupted sutures (6/0 and/or 7/0 polypropylene [Ethicon, Johnson & Johnson, Somerville, USA]) at the level of the papillae. Simple interrupted sutures were performed at the level of the vertical incisions, if any (7/0 polypropylene [Ethicon, Johnson & Johnson, Somerville, USA]) [18]. The post‐operative regimen is reported in Appendix S1 and in the original publication [18].

### 2.4 Study Outcomes

The current study aimed at evaluating the changes within ultrasonographic tissue perfusion occurring over 6 months following root coverage procedure with either CCMX + saline or CCMX + rhPDGF. Correlations between early tissue perfusion and the final clinical, volumetric, and patient‐reported outcomes were also explored. Secondary outcomes of the study involved the assessment of the expression of wound healing biomarkers.

### 2.5 Ultrasonographic Imaging

Ultrasonographic equipment and set up have been previously described in detail [34, 43]. Briefly, an ultrasound imaging device (ZS3, Zonare/Mindray, Mountain View, CA, USA) was utilized with a 24 MHz (64 μm axial image resolution) and miniature‐sized (30 × 18 × 12 mm) transducer (L30‐8, Zonare/Mindray, Mountain View, CA, USA) to generate ultrasound images at baseline (prior to the initiation of the surgical procedure) and 2 weeks, 3 months, and 6 months following surgery. Single image frames (“still” images) were obtained in brightness mode (“B‐mode”) at the mid‐facial, interproximal aspect, and transverse aspect of the site of interest. B‐mode images were saved in the digital imaging and communications in medicine (DICOM) format.

### 2.6 Doppler Ultrasonographic Tissue Perfusion

After obtaining the B‐mode images, tissue perfusion was assessed using the color doppler velocity (CDV) and power doppler imaging (PDI). CDV and PDI are imaging modalities in which the B‐mode is overlaid with additional color pixels that represent detected blood flow. CDV allows to detect mean velocity of blood flow within vessels through color coding, based on the scattered signal produced by moving red blood cells that results in a change in frequency of the reflected sounds waves that are received by the ultrasound transducer [29]. Color flow provides information on blood flow velocity direction and velocity magnitude, with hues of red and blue colors that are assigned to image pixels based on these two parameters. Blood flow moving towards the transducer is conventionally displayed in red, while blood flowing away from the transducer is usually indicated in blue. PDI is also based on the detection of phase shift changes of the received ultrasound signal, and it provides information on blood flow quantity that is related to the number of contributing red blood cells that scatter the transmitted acoustic wave. PDI displays in a single‐hue red color the amount of blood flowing within the lumens in the field of view [24, 34, 36].

Six second cine loops of CDV and PDI modalities capturing at least 5 cardiac cycles were recorded at the midfacial, interproximal, and transverse aspect of the sites of interest. These cine loop videos were saved and exported as DICOM files. Tissue perfusion analysis was performed using a software package (PixelFlux, version 2018, Chameleon‐Software, Germany). For cine loops obtained at the midfacial aspect, the region of interest (ROI) was defined within the soft tissue as the area between the gingival margin and extending 7 mm apically to this landmark. For cine loops depicting the interproximal aspect, the ROI had the same landmarks (gingival margin and a point extending 7 mm apical to this reference) but also extended interproximally at the coronal aspect (papilla). For cine loops capturing tissue perfusion at the transverse aspect, the area encompassing the mesial, midfacial, and distal soft tissue of the tooth of interest was defined as the ROI [35]. After importing the DICOM files containing the cine loops obtained in CDV modality and after tracing the ROI, the software automatically computed the following dynamic tissue perfusion measurements (DTPMs), as previously described [33, 36, 44, 45, 46]:

- Mean perfusion relief intensity (pRI) and mean perfused area (pA) were obtained from the “perfusion relief” function of the software, which provides a visual impression of the vasculature depicting the local distribution and intensity of the perfusion within the ROI
- Flow velocity red (*V*_red_), flow velocity blue (*V*_blue_), flow velocity mix (*V*_mix_), and total flow velocity (*V*_tot_) where flow velocity corresponds to the color hue of the pixels within the selected ROI. *V*_red_ depicts the tissue perfusion occurring towards the transducer only, and *V*_blue_ captures the blood flowing away from the transducer only, while *V*_mean_ depicts the average between *V*_red_ and *V*_blue_.
- Flow intensity (FI) was calculated by the software with the formula:
$$\mathrm{FI}\;\left(\mathrm{cm}/s\right)=\frac{\text{velocity}\;\left(\mathrm{cm}/s\right)\times A\;\left({\mathrm{cm}}^{2}\right)}{A_{\mathrm{ROI}}\;\left({\mathrm{cm}}^{2}\right)}$$
  where velocity corresponds to the color hue of the pixels within the selected ROI, “A” is the mean perfused area determined by the number of perfused pixel within the ROI, and A_ROI_ is the total area of the ROI. FI was calculated as FI_red_, depicting the FI of the blood flowing towards the transducer only, FI_blue_, capturing the perfusion intensity of the blood flowing away from the transducer only, FI_mean_, calculated as the average between FI_red_ and FI_blue_, while FI_tot_ was obtained as the sum of FI_red_ and FI_blue_. Flow intensity (PDI FI) was also calculated on the cine loops obtained in PDI modality.

**FIGURE 1:**
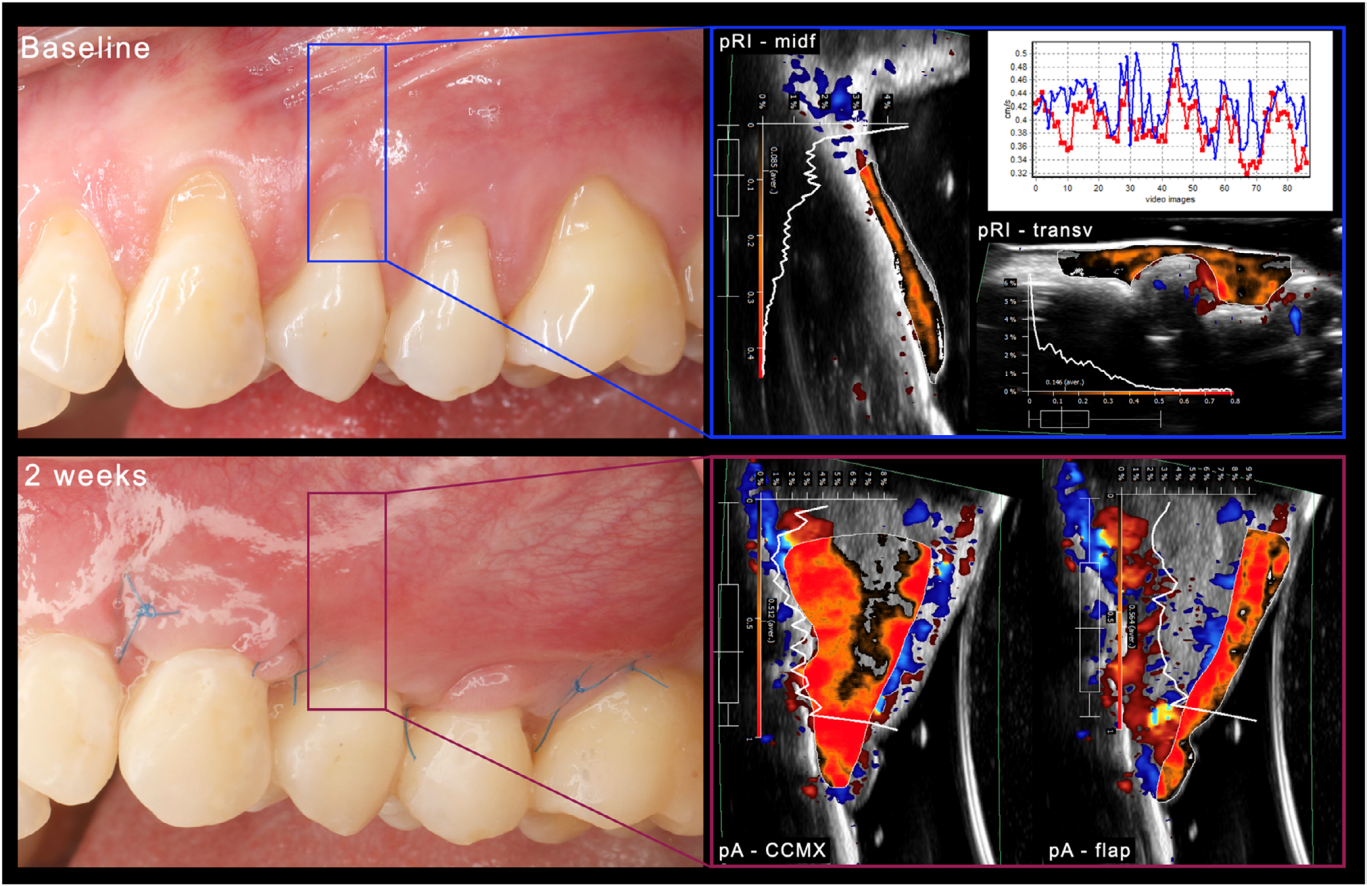
Ultrasonographic dynamic tissue perfusion assessment at baseline and 2 weeks after root coverage procedure with a xenogeneic cross‐linked collagen matrix (CCMX). The figures on the right side of the panel depict the quantification of the mean perfusion relief intensity (pRI) at the midfacial (“midf”) and transverse (“transv”) aspects. The plot illustrates FI_red_ and FI_blue_. In the lower part of the panel the mean perfused area (pA) of the graft and flap at the 2‐week time point are shown. pRI and pA refer to the number of pixel within the region of interest (graft) that relate to the intensity of the respective value. Areas with zero intensity/perfusion within the ROI are shown in black, while areas with half of the maximum intensity/perfusion and maximum intensity/perfusion are visualized in orange and red, respectively.

At the 2‐week time point, it was possible to discriminate between the grafted area and the flap at the midfacial ultrasound scans, and therefore, the following DTPMs were performed for the graft (CCMX) and the flap: pRI, pA, *V*_mean_, FI_red_, FI_blue_, FI_mean_, and FI_tot_ (Figure 1).

### 2.7 Operator Calibration

A single operator (T.N.), masked to the treatment allocation, who had also not taken part in the surgical portion of the clinical trial, performed all the tissue perfusion analyses. The operator had received extensive training in dental ultrasonography prior to the beginning of the analysis, with the calibration that consisted of two repeated analysis of the DTPMs of interest, performed one week apart, on ultrasonographic scans of 10 subjects who had not participated in the present study. The *K* coefficient was ≥ 0.85 for all the DTPMs.

### 2.8 Clinical, Volumetric, and Patient‐Reported Outcome Measures

The following clinical measurements were performed at baseline and 6 months after the surgery by a single masked and calibrated examiner, as previously described in detail: recession depth, mean root coverage (mRC), complete root coverage (CRC), probing depth, clinical attachment level, and keratinized tissue width. Gingival thickness (GT) was evaluated on midfacial ultrasound scans that were imported as DICOM files in a public‐domain software package (Horos, version 4.0.0, Horos Project) for evaluating gingival thickness at 1.5‐ and 3‐mm reference points from the gingival margin. GT gain was obtained by assessing the difference between GT gain at 6 months compared to baseline. Volumetric changes from baseline to 6 months (Vol gain) were assessed by superimposing the digital impressions obtained at baseline with those collected at 6 months, utilizing an image analysis software (GOM Inspect, GOM, Germany). Additional information on operator masking and calibration regarding these outcomes are reported in the original publication [18]. Patient‐reported outcome measures (PROMs) included post‐operative morbidity, assessed using a questionnaire that included a 100‐mm visual analogue scale (VAS) for each of the 15 post‐operative days and time to recovery, that was calculated as the time required to reach a VAS < 10 [18, 42].

### 2.9 Biofluid Collection and Processing

GCF was collected from the midfacial aspect of the experimental sites and untreated sites at baseline (the day of the surgery, prior to performing local anesthesia) and at 7, 14, 30, and 90 days after the intervention. Contralateral teeth were prioritized to be selected as untreated sites; however, when the experimental area was located in the second or fifth sextant, the correspondent teeth in the opposite arch were chosen as untreated sites. Methylcellulose paper strips (Periopaper, Oraflow, Smithtown, NY, USA) were used for GCF collection. After air‐drying the area of interest, the paper strip was gently inserted, until slight resistance was felt, into the sulcus at the midfacial aspect, for 30 s [47]. Each strip was placed in an individually labeled plastic vial and stored in a −80°C freezer until the analysis was performed. GCF samples were processed all at once after the conclusion of the clinical portion of the study. A 20‐μL extraction solution (10 mg/mL aprotinin, 1 mM phenylmethylsulfonyl fluoride, and 0.1% human serum albumin in phosphate‐buffered saline) was pipetted onto the cellulose portion of each GCF strip that was secured at the top of a polystyrene culture tube (12 × 75 mm) using a cap to keep the strip in place. Then, the tubes were centrifuged at 2000 rpm at 4°C for 5 min. Each strip was washed and centrifuged five times to yield a total elution volume of 100 μL. Samples were added to custom human antibody arrays and then scanned and analyzed according to the manufacturer's protocol (RayBiotech Life Inc., Norcross, GA, USA), as previously described [47, 48, 49]. The biomarkers selected for the array included: (i) angiogenin (ANG), (ii) basic fibroblast growth factor (bFGF), (iii) interleukin‐1β (IL‐1β), (iv) interleukin‐6 (IL‐6); (v) interleukin‐10 (IL‐10); (vi) platelet‐derived growth factor‐BB (PDGF‐BB); (vii) transforming growth factor β1 (TGF β1); (viii) tissue inhibitor of metalloproteinases‐2 (TIMP‐2); (ix) tumor necrosis factor‐α (TNF‐α); and (x) vascular endothelial growth factor (VEGF).

### 2.10 Statistical Analysis

Means and standard deviations (SD) were calculated for describing continuous variables. Longitudinal regression models using generalized estimation equations (GEE) were conducted to assess changes of the tissue perfusion parameters over time according to the treatment group (CCMX + saline vs. CCMX + rhPDGF). Wald's Chi‐Squared test was used to conclude about main effects and interactions. Pairwise comparisons were adjusted by Bonferroni's correction. Regression models using GEE were performed to evaluate relationships between ultrasonographic tissue perfusion parameters and clinical outcomes. The significance level used in the analysis was 5% (*α* = 0.05). The analyses were performed by an independent biostatistician, who had not taken part in the clinical portion of the study or data collection.

## 3 Results

### 3.1 Study Participants

Thirty subjects (mean age 38.4 ± 11.5 years, 19 females, 11 males), each contributing with 2–5 MAGRs, were randomized and allocated to receive either the test (CCM + rhPDGF, 15 subjects for a total of 47 sites) or control (CCMX with saline, 15 subjects for a total of 44 sites) treatment. Patient characteristics and baseline clinical measurements are depicted in Table 1. All subjects completed the appointment visits over 6 months.

**TABLE 1:** Study population and baseline characteristics of the study sites. No statistically significant differences were observed between the two groups at baseline.

Parameter	CCMX + saline	CCMX + rhPDGF
Age (mean ± SD) (years)	40.9 ± 12.3	36.0 ± 11.0
Females (*N*)/(%)	8/53.3	11/73.3
Smokers (≤ 10 cig/day) (*N*)	1	0
Total Sites (*N*)	44	47
Sites with NCCLs (*N*)	7	6
Sites in which the CEJ was reconstructed (*N*)	7	6
Rec depth (mean ± SD) (mm)	3.05 ± 1.21	2.87 ± 0.78
PD (mean ± SD) (mm)	1.46 ± 0.61	1.39 ± 0.54
CAL (mean ± SD) (mm)	4.51 ± 1.59	4.27 ± 0.89
KTW (mean ± SD) (mm)	2.10 ± 1.28	2.48 ± 0.87
GT (mean ± SD) (mm)	0.84 ± 0.27	0.92 ± 0.26

Abbreviations: CAL, clinical attachment level; CCMX, cross‐linked xenogeneic collagen matrix; GT, gingival thickness; KTW, keratinized tissue width; NCCLs, non‐carious cervical lesion; PD, pocket depth; Rec, recession; rhPDGF, recombinant human platelet‐derived growth factor; SD, standard deviation.

### 3.2 Blood Flow Changes Over Time Occurring Within the Whole Soft Tissue Area

The longitudinal regression analysis assessing the pattern of tissue perfusion changes at the midfacial aspect over 6 months did not find significant differences between test and control groups in terms of CDV pRI, CDV pA, CDV *V*_mean_, CDV *V*_red_, CDV *V*_blue_, CDV FI_tot_, CDV FI_mean_, CDV FI_red_, CDV FI_blue_, and PDI FI. Bonferroni's test pairwise comparison revealed that, although not statistically significant, the p‐value estimating the difference between test and control sites for CDV *V*_red_ at 2 weeks was approaching statistical significance (*p* = 0.078) (Figure S2).

The longitudinal regression analysis assessing the pattern of tissue perfusion changes at the interproximal aspect over 6 months did not find statistically significant differences between test and control groups in terms of CDV pRI, CDV *V*_red_, CDV FI_tot_, CDV FI_mean_, CDV FI_red_, CDV FI_blue_, and PDI FI. Test and control sites exhibited a statistically significant different pattern in terms of changes of CDV pA (estimated coefficient of −0.005) (95% CI [−0.009, −0.001], *p* = 0.025) and *V*_blue_ over time (estimated coefficient of 0.009) (95% CI [0.002, 0.015], *p* = 0.008). Although not statistically significant, the difference between test and control sites for CDV V_mean_ was approaching statistically significance (estimated coefficient of 0.006) (95% CI [−0.001, 0.012], *p* = 0.073) (Figure S3).

The longitudinal regression analysis assessing the pattern of tissue perfusion changes at the transverse aspect over 6 months did not find statistically significant differences between test and control groups in terms of CDV pRI, CDV pA, CDV *V*_mean_, CDV *V*_red_, CDV *V*_blue_, CDV FI_tot_, CDV FI_mean_, CDV FI_red_, and PDI FI. On the other hand, a statistically significant difference was found in the pattern of changes of CDV FI_blue_ between test and control sites (estimated coefficient of −0.002) (95% CI [−0.003, 0.000], *p* = 0.021) (Figure S4).

### 3.3 Dynamic Tissue Perfusion Occurring Within the Graft and the Flap at 2 Weeks

#### 3.3.1 Mean Perfusion Relief Intensity (pRI)

**FIGURE 2:**
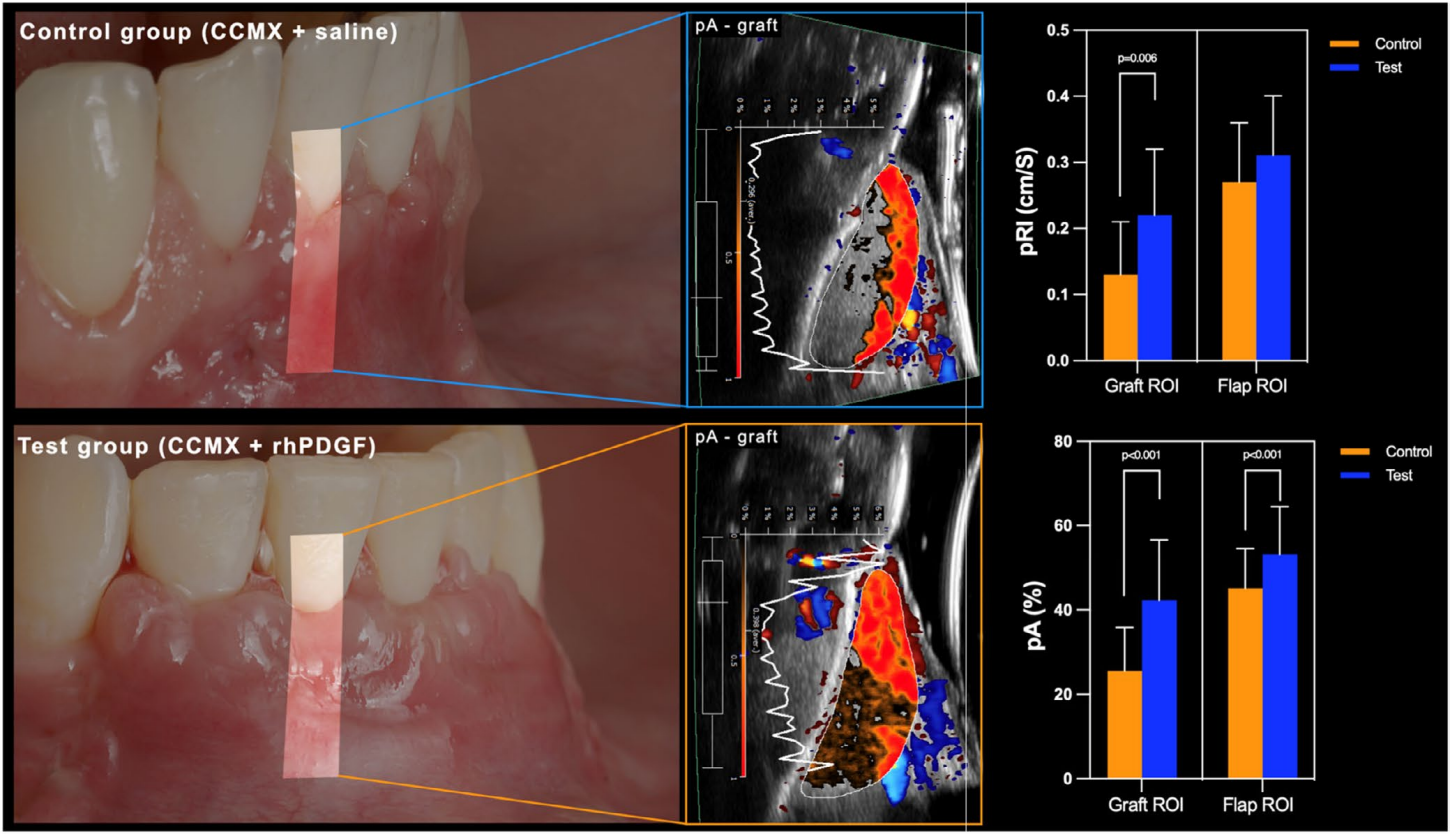
Dynamic tissue perfusion assessment at 2 weeks at sites treated with CCMX loaded with saline or with rhPDGF. The right panels illustrate the determination of the region of interest (ROI) at the level of the graft for the calculation of the mean perfused area (pA), and the plots depicting mean perfusion relief intensity (pRI), and pA at the graft and flap ROIs within the two groups at 2 weeks. Figure readapted and reproduced with permission John Wiley & Sons [19].

Regression analysis demonstrated that pRI was significantly different at test and control sites at 2 weeks (estimated coefficient of 0.092) (95% CI [0.038, 0.147], *p* = 0.001). In particular, pRI was found to be significantly different when measured at the graft region compared to the flap area (estimated coefficient of 0.138) (95% CI [0.097, 0.179], *p* < 0.001) in both groups. Bonferroni's test revealed a statistically significant difference between the test and control for pRI at the graft ROI only (*p* = 0.006). The pRI at the graft ROI at sites treated with CCMX + rhPDGF was 0.22 ± 0.10 cm/s, while at sites allocated to CCMX alone, the respective pRI was 0.13 ± 0.08 cm/s (*p* = 0.006) (Figure 2). No significant differences were found at the level of the flap (0.31 ± 0.09 cm/s and 0.27 ± 0.09 cm/s for the test and control group, respectively, *p* > 0.05).

#### 3.3.2 Mean Perfusion Relief Area (pA)

pA was significantly different between test and control sites (estimated coefficient of 16.72) (95% CI [8.71, 24.73], *p* < 0.001), and between flap and graft ROIs (estimated coefficient of 19.52) (95% CI [14.32, 24.73], *p* < 0.001). Bonferroni's test revealed a statistically significant difference between test and control sites in terms of pA both at the graft ROI (*p* < 0.001) and also at the flap ROI (*p* = 0.033). Test sites exhibited a pA of the graft of 42.25% ± 14.34%, while the same parameter at control sites was 25.53% ± 10.30% (*p* < 0.001). The test group also showed a superior pA within the flap ROI compared to the control group (53.10 ± 11.42 vs. 45.06 ± 9.43, respectively, *p* = 0.033) (Figure 2).

#### 3.3.3 Mean Flow Velocity (*V*_mean_)

No differences were found between test and control groups in terms of *V*_mean_, while a statistically significant *V*_mean_ was observed at the graft ROI compared to the flap ROI (estimated coefficient of 0.079) (95% CI [0.034, 0.124], *p* = 0.001). Bonferroni's test revealed a statistically significant difference between *V*_mean_ at the graft vs. flap both at control sites (*p* = 0.003) and also at test sites (*p* = 0.002) (Table 2).

**TABLE 2:** Dynamic tissue perfusion measurements at the graft and flap region of interest 2 weeks after root coverage procedure.

DTPM	Control sites (15 Subjects, 44 sites)		Test sites (15 Subjects, 47 sites)	
	Graft ROI (mean ± SD)	Flap ROI (mean ± SD)	Graft ROI (mean ± SD)	Flap ROI (mean ± SD)
pRI (cm/s)	0.13 ± 0.08^a^, ^b^	0.27 ± 0.09	0.22 ± 0.10^a^, ^b^	0.31 ± 0.09
pA (%)	25.53 ± 10.30^a^, ^b^	45.06 ± 9.43^a^, ^b^	42.25 ± 14.34^a^, ^b^	53.10 ± 11.42^a^, ^b^
*V*_mean_ (cm/s)	0.48 ± 0.13^b^	0.56 ± 0.12^b^	0.49 ± 0.09^b^	0.55 ± 0.08^b^
FI_red_ (cm/s)	0.05 ± 0.05^b^	0.13 ± 0.08^b^	0.09 ± 0.06	0.11 ± 0.08
FI_blue_ (cm/s)	0.06 ± 0.04^b^	0.11 ± 0.07^a^, ^b^	0.09 ± 0.06^b^	0.17 ± 0.09^a^, ^b^
FI_mean_ (cm/s)	0.06 ± 0.03^a^, ^b^	0.12 ± 0.05^b^	0.09 ± 0.04^a^, ^b^	0.14 ± 0.04^b^
FI_tot_ (cm/s)	0.11 ± 0.07^a^, ^b^	0.25 ± 0.10^b^	0.18 ± 0.09^a^, ^b^	0.29 ± 0.09^b^

*Note:* FI_blue_, flow intensity “blue”, meaning the intensity of the tissue perfusion flowing away from the transducer; FI_mean_, mean flow intensity mix, meaning the average between FI_red_ and FI_blue_; FI_red_, flow intensity “red”, meaning the intensity of the tissue perfusion flowing towards the transducer; FI_tot_, total flow intensity, meaning the sum of FI_red_ and FI_blue_; pA, mean perfused area; *V*_mean_, mean velocity of the flow, obtained as the sum of *V*_red_ and *V*_blue_.

Abbreviations: DTPM, dynamic tissue perfusion measurements; pRI, perfusion relief intensity; ROI, region of interest; SD, standard deviation.

^a^ Statistically significant difference between test and control group for this DTPM at this ROI.

^b^ Statistically significant difference between graft ROI and flap ROI within the same treatment group.

#### 3.3.4 Flow Intensity Red (FI_red_ )

FI_red_ was significantly different between test and control sites (estimated coefficient of 0.037) (95% CI [0.007, 0.068], *p* = 0.016), and between graft and flap ROIs (estimated coefficient of 0.083) (95% CI [0.055, 0.111], *p* < 0.001). FI_red_ of the graft was statistically significantly different from FI_red_ of the flap at control sites only (*p* < 0.001) but not at test sites (*p* = 0.584) (Table 2).

#### 3.3.5 Flow Intensity Blue (FI_blue_ )

FI_blue_ was significantly different between test and control sites (estimated coefficient of 0.033) (95% CI [0.005, 0.061], *p* = 0.019) and between graft and flap ROIs (estimated coefficient of 0.053) (95% CI [0.03, 0.08], *p* < 0.001). Bonferroni's test revealed a statistically significant difference between test and control groups in terms of FI_blue_ at the flap ROI (*p* = 0.035) but not at the graft ROI (*p* = 0.114) (Table 2).

#### 3.3.6 Mean Flow Intensity (FI_mean_ )

FI_mean_ was significantly different between test and control sites (estimated coefficient of 0.035) (95% CI [0.012, 0.059], *p* = 0.003) and between graft and flap ROIs (estimated coefficient of 0.068) (95% CI [0.047, 0.090], *p* < 0.001). Bonferroni's test revealed a statistically significant difference between test and control groups in terms of FI_mean_ at the graft ROI (*p* = 0.021) but not at the flap ROI (*p* = 0.58). On the other hands, FI_mean_ of the graft was significantly different from FI_mean_ of the flap at the test and control sites (*p* < 0.001 for both groups) (Table 2).

#### 3.3.7 Total Flow Intensity (FI_tot_ )

FI_tot_ was significantly different between test and control sites (estimated coefficient of 0.071) (95% CI [0.023, 0.118], *p* = 0.003) and between graft and flap ROIs (estimated coefficient of 0.136) (95% CI [0.093, 0.179], *p* < 0.001). Bonferroni's test revealed a statistically significant difference between test and control groups in terms of FI_tot_ at the graft ROI (*p* = 0.021) but not at the flap ROI (*p* = 0.58). On the other hands, FI_tot_ of the graft was significantly different from FI_tot_ of the flap at the test and control sites (*p* < 0.001 for both groups) (Table 2).

#### 3.3.8 Correlations Between DTPMs and Clinical, Volumetric, and Patient‐Reported Outcomes

**FIGURE 3:**
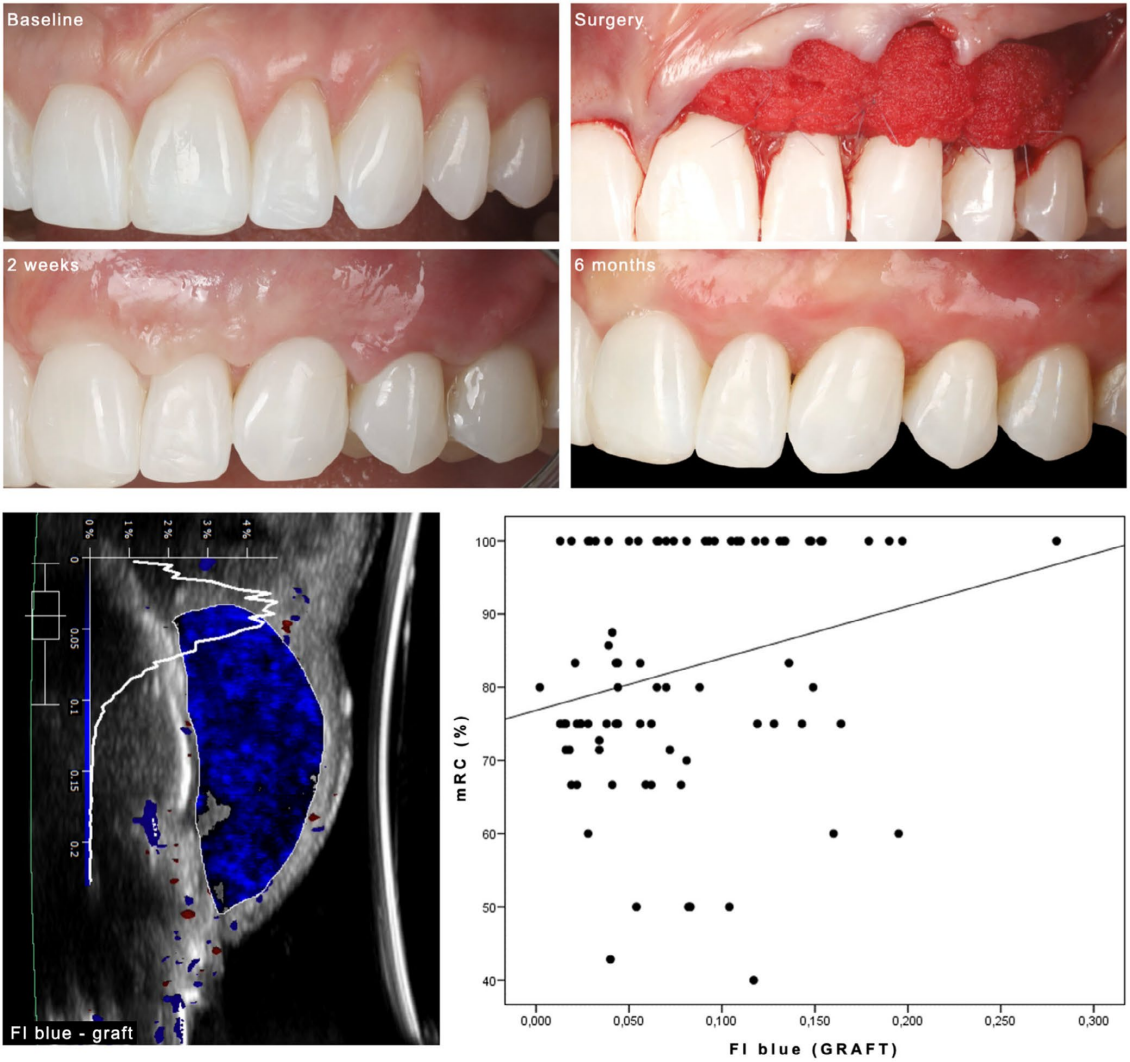
The upper panel illustrates a case of MAGRs in the maxilla treated with a CAF in combination with a CCMX saturated with rhPDGF. The lower figures depict the assessment of FI_blue_ within the graft region of interest at 2 weeks, and the plot showing the direct correlation between FI_blue_ at the level of the graft and the final mean root coverage (mRC).

Regression analysis demonstrated that FI_blue_ at the graft ROI was significantly associated with mRC (estimated coefficient of 71.3) (95% CI [18.2, 124.4], *p* = 0.008). In other words, increased values of FI_blue_ at the level of the CCMX at 2 weeks involved increased mRC outcomes at 6 months (Figure 3). Similarly, regression analysis demonstrated that FI_blue_ at the graft ROI was significantly associated with CRC (OR of 0.001) (95% CI [0.001, 0.006], *p* = 0.003). At 2 weeks, the mean FI_blue_ at the graft ROI at sites that obtained CRC at the end of the study was 0.103 cm/s, while the same parameters at sites that did not obtaine CRC was, on average, 0.063 cm/s.

**FIGURE 4:**
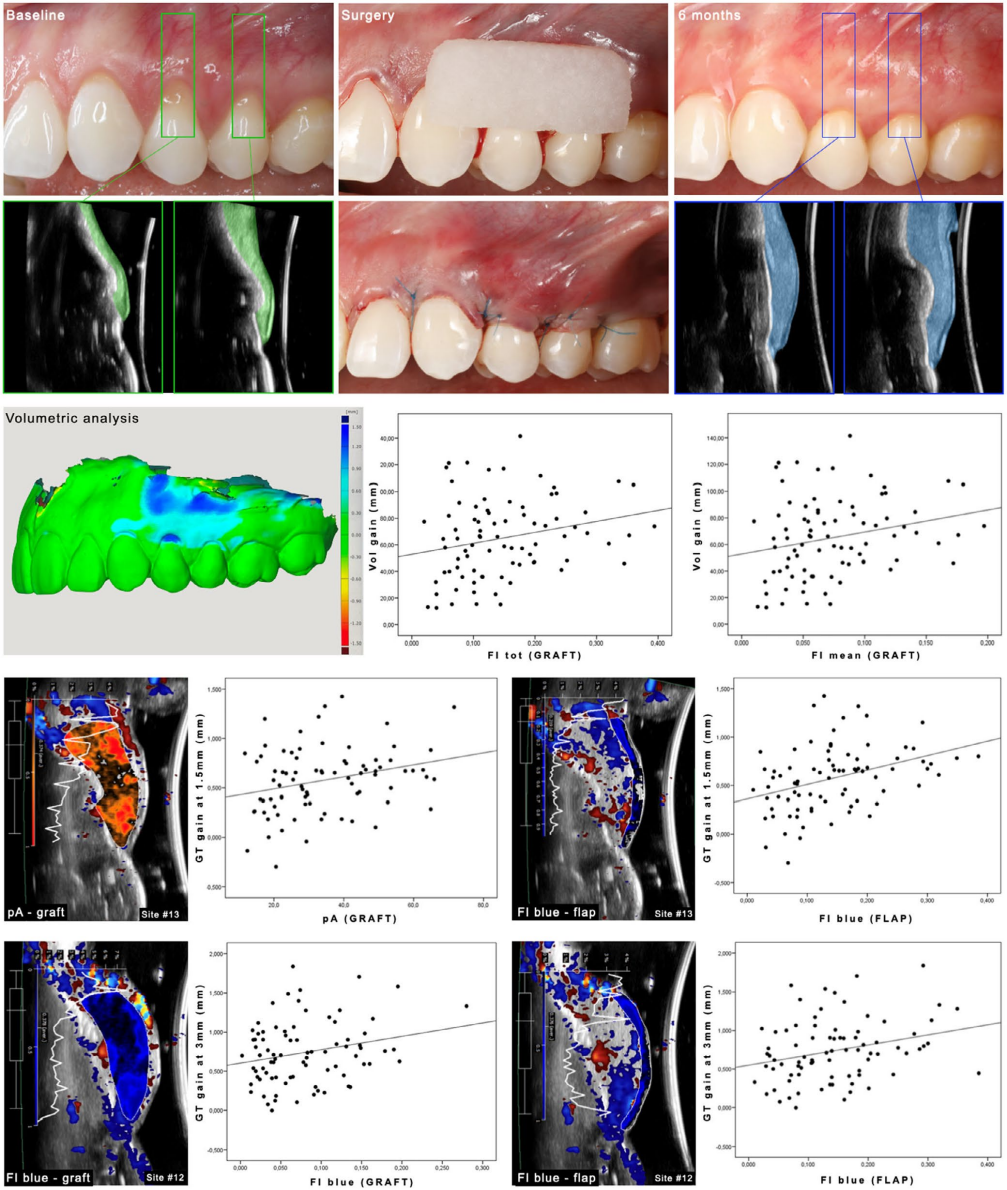
The upper panel shows the baseline clinical and ultrasonographic views as well as the intrasurgical photos and the outcomes at 6 months. The soft tissue of the two premolars at baseline was highlighted in green, while the augmented soft tissue at 6 months was depicted in light blue. The respective volumetric analysis obtained from the superimposition of the digital models at baseline and 6 months is reported below, together with the plot depicting the correlation between FI_tot_, FI_mean_, and final volume gain (Vol gain). The lower panels exhibit the assessment of pA and FI_blue_ at the level of the graft or the flap, with the respective plot highlighting the correlations of these parameters with gingival thickness (GT) gain at 6 months.

Linear regression demonstrated that Vol gain at 6 months was statistically significantly correlated with FI_tot_ of the graft at 2 weeks (estimated coefficient of 82.4) (95% CI [7.66, 157.2], *p* = 0.031) and FI_mean_ of the graft at 2 weeks (estimated coefficient of 165.6) (95% CI [15.5, 315.8], *p* = 0.031). In other words, the higher these parameters at the graft ROI, the greater the final Vol gain (Figure 4).

pA of the graft was found to be significantly associated with GT gain at the 1.5 mm‐reference point (estimated coefficient of 0.006) (95% CI [0.001, 0.011], *p* = 0.019). GT gain at the 1.5 mm‐level was also found to be significantly associated with pA of the flap (estimated coefficient of 0.006) (95% CI [0.002, 0.011], *p* = 0.010) and FI_blue_ of the flap (estimated coefficient of 1.49) (95% CI [0.82, 2.15], *p* < 0.001). Similarly, GT measured at 3 mm was found to be significantly associated with FI_blue_ at the graft ROI (estimated coefficient of 1.71) (95% CI [0.52, 2.91], *p* = 0.005) and FI_blue_ at the flap ROI (estimated coefficient of 1.31) (95% CI [0.38, 2.24], *p* = 0.006) (Figure 4).

There were no associations between early DTPMs and patient‐reported pain after surgery. However, a moderately weak association was noted between pRI at the graft ROI and time to recovery (*r* = −0.38, *p* = 0.04) and between pA at the graft ROI and time to recovery (*r* = −0.43, *p* = 0.02).

### 3.4 Biomarker Expressions Over 3 Months

**FIGURE 5:**
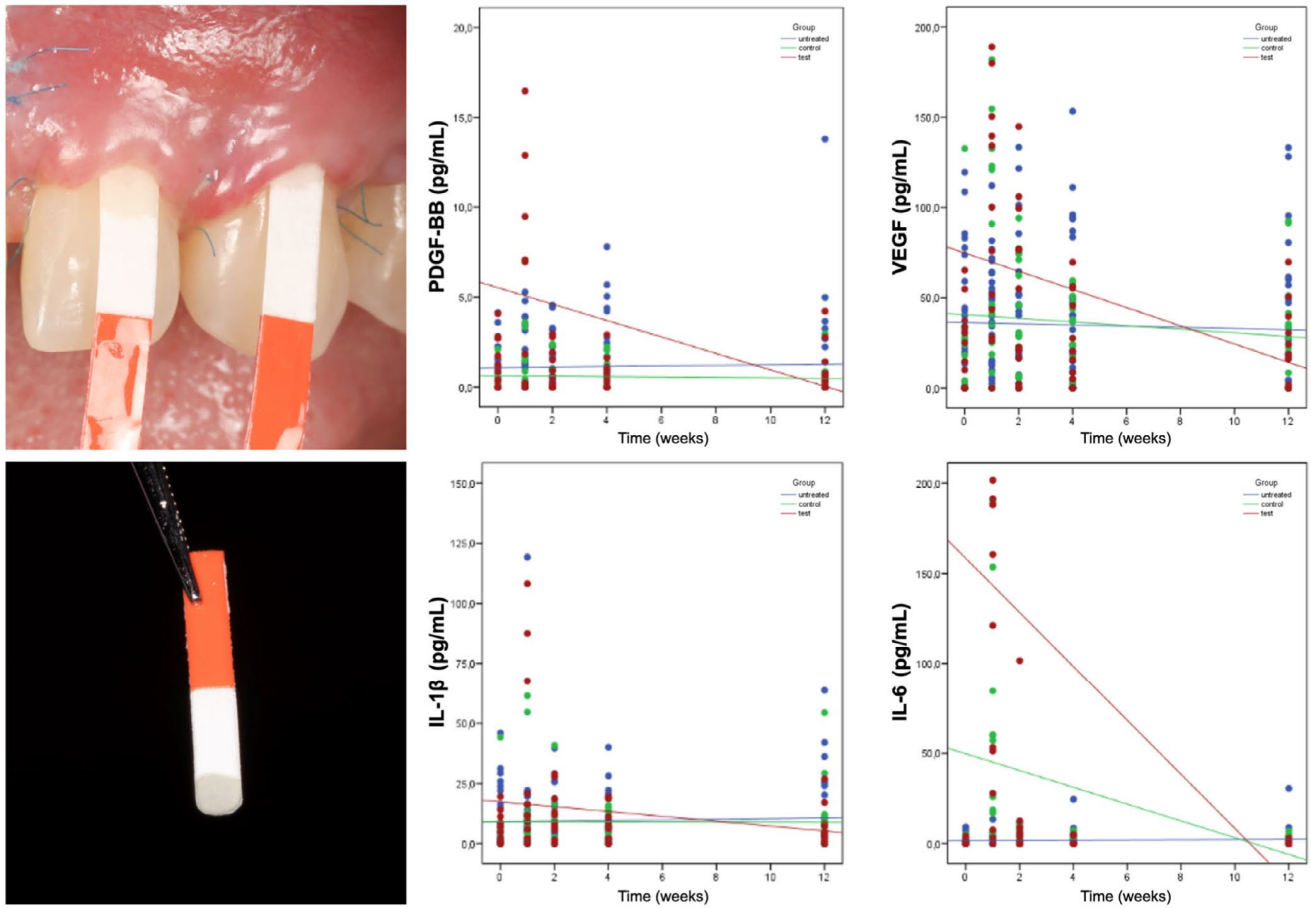
Gingival crevicular fluid collection and scatter plots depicting the slopes related to the changes in biomarker levels at the test, control, and untreated sites.

The expression of ANG, bFGF, IL‐1β, IL‐6, IL‐10, PDGF‐BB, TGF β1, TIMP‐2, TNF‐α, and VEGF at the control, test, and untreated sites is reported in detail in Figure 5 and in Appendix S1.

The regression analysis revealed a statistically significant different expression of IL‐1β (estimated coefficient of −0.98) (95% CI [−1.94, −0.03], *p* = 0.043), PDFG‐BB (estimated coefficient of −0.44) (95% CI [−0.74, −0.14], *p* = 0.003), and VEGF (estimated coefficient of −4.04) (95% CI [−7.12, −0.95], *p* = 0.010) between test and control sites over 3 months. Sites treated with CCMX + rhPDGF demonstrated a statistically significant higher expression of ANG (estimated coefficient of −20.3) (95% CI [−36.2, −4.4], *p* = 0.012), IL‐1β (estimated coefficient of −1.13) (95% CI [−1.93, −0.34], *p* = 0.005), IL‐6 (estimated coefficient of −15.0) (95% CI [−26.0, −4.07], *p* = 0.007), PDGF‐BB (estimated coefficient of −0.47) (95% CI [−0.76, −0.18], *p* = 0.02), and VEGF (estimated coefficient of −4.71) (95% CI [−7.40, −2.03], *p* = 0.001) compared to untreated sites. Sites allocated to CCMX + saline exhibited a significantly greater expression of IL‐6 (estimated coefficient of −4.69) (95% CI [−8.46, −0.93], *p* = 0.014) than untreated sites (Figures S5 and S6). Correlations between the investigated biomarkers at different time points are reported in Table S2.

### 3.5 Early Wound Healing Biomarker Expression and Patient‐Reported Morbidity

No significant associations were found between the expression of the biomarkers of interested at baseline and at 1 week with patient‐reported post‐operative pain nor with the final clinical outcomes. On the other hand, a significant inverse correlation was demonstrated between the levels of PDGF‐BB at 1 week and the patient‐reported time to recovery (correlation coefficient *r* = −0.41, *p* = 0.029). In other words, shorter times to recovery were observed in subjects exhibiting high PDGF‐BB values at 1 week.

## 4 Discussion

Patient perception of surgical procedures and PROMs have progressively become of paramount importance in dentistry [50, 51, 52]. Patient‐centered, minimally invasive, approaches for the treatment of gingival recessions often involve novel surgical techniques, site‐specific use of autogenous grafts, graft substitutes, biologics, and their combinations [53, 54, 55, 56]. As recent evidence advocates the use of graft substitutes as scaffolds for biologic agents [18, 57, 58], even though the mechanism of action of these combination therapies has not been demonstrated in a clinical setting, the present manuscript aimed at using HFUS and biomarkers to characterize tissue perfusion and wound healing following soft tissue grafting either with CCMX alone or saturated rhPDGF.

HFUS employs the acoustic signal phase‐shift effects resulting from the movement of red blood cells in the vessels to visualize blood flow of the graft, the flap, or the whole region [24, 29, 36]. Specific software allows to quantify tissue perfusion that can be expressed in terms of intensity of the blood flow, velocity of the blood flow, and mean area of the ROI that is perfused, with the possibility of further discriminating tissue perfusion outcomes based on their direction [33, 36, 44, 45, 46]. The primary goal of the present manuscript was to investigate the blood flow changes occurring at the treated sites over 6 months, and to evaluate the early tissue perfusion occurring at the level of the graft and the flap within the two groups.

Overall, the control and test sites exhibit a similar pattern for most of the investigated tissue perfusion outcomes over 6 months. The main differences between the two groups were observed at the 2‐week time points when the tissue perfusion of the graft and the flap were assessed independently. CCMXs loaded with rhPDGF exhibited statistically significant higher pRI, pA, FI_mean_, and FI_tot_ compared to CCMXs that were not saturated with the growth factor. In particular, pRI and pA assessed at the level of the graft were approximately 1.7 times higher in the test group compared to the control group, highlighting the effect of rhPDGF on the vascularization of the matrix within the first 2 weeks. PDGF has been shown to upregulate angiogenesis by recruiting perivascular cells that induce formation and stabilization of new vessels [59]. Experimental models demonstrated that the number and proliferation rate of perivascular cells in the small blood vessels is reduced in the absence of PDGF [60]. Stimulating the activity of PDGF‐β receptors with PDGF promotes the induction of VEGF, which further contributes to pericyte coating and vasculogenesis [60, 61]. In addition, it was observed that these properties of PDGF are intensified by specific molecules of the extracellular matrix, such as collagens and heparin, with the PDGF‐heparin‐collagen complex that further stimulates fibroblasts activity, cell migration, and angiogenesis [60, 62]. These mechanisms of action of the growth factor at early time points are in line with our ultrasonographic findings, showing a superior vascularization (at the 2‐week time point) of the grafts that were loaded with rhPDGF. The importance of graft vascularization has been recently highlighted in a study from our group, where the early tissue perfusion of connective tissue grafts at implant sites was found to be directly correlated with the volume gains obtained at 1 year, suggesting that the process and timing of revascularization of soft tissue grafts may contribute to their volume stability or shrinkage [36].

Regression analysis showed that tissue perfusion of the CCMXs, in terms of FI_blue_, at 2 weeks was significantly and directly correlated to mRC and CRC at 6 months. This finding further supports the speculation that high vascularization of the graft at early time points could lead to a faster healing, with a shorter inflammation phase, and consequent higher stability of the gingival margin and the soft tissue volume [36]. Other DTPMs at the 2‐week time point, assessed at the level of the graft, were found to predict volume gain and GT gain at 6 months. It is interesting to highlight that these parameters were FI_blue_, FI_tot_, and FI_mean_, while no correlations were found with FI_red_, suggesting that the most crucial blood supply at this time point is the one flowing away from the ultrasound transducer, from the most superficial region of the soft tissue towards the bone/root, which is displayed in blue in the ultrasound scans. This observation is in line with a previous study assessing the revascularization of autogenous connective tissue graft at early time points [36].

The correlation between early tissue perfusion of the graft and the flap with the final volume and GT gains has important clinical implications, as the role of GT, and overall, the gingival phenotype, 6 months after root coverage procedures, has shown to play a key role on the stability of the gingival margin over 10 years, regardless of the treatment performed [6]. It can be speculated that grafts and flaps that are properly manipulated and stabilized may be the ones healing with the high tissue perfusion at early time points, providing the greater volume and GT gains at 6 months, that, together with other factors, such as the keratinized tissue width, initial recession area, non‐carious cervical lesions, and patient compliance with atraumatic toothbrushing, among others [4, 6, 63, 64, 65], could contribute to the stability of the root coverage outcomes over time. This assumption needs to be assessed in future long‐term studies.

It was also observed that pRI and pA at the level of the CCMX at 2 weeks were inversely associated with recovery time. An increase in tissue perfusion of grafts at 2 weeks may be associated with advanced healing phases, lower level of inflammation, and high concentrations of proteins contributing to wound healing, which may explain the faster time to recovery perceived by patients.

Previous studies have explored the evaluation of the expression of extracellular matrix molecules from the GCF as a non‐invasive method to assess wound healing events associated with different soft tissue grafting procedures [47, 66, 67]. Our findings revealed that the expression of IL‐1β, PDFG‐BB, and VEGF at sites allocated to rhPDGF was significantly different compared to the expression of these molecules at sites treated without the growth factor. VEGF is a pro‐angiogenic factor which is involved in various signaling pathways related to the vascular system and early wound healing [47, 68]. A recent publication demonstrated that VEGF levels 7 days after performing connective tissue graft at implant sites were directly associated with the final gain in mucosal thickness [49]. TIMP‐2 was also found to be correlated with the final amount of soft tissue coverage at implant sites [49]. Skurska et al. investigated the concentration of metalloproteinases‐1 (MMP‐1) and ‐8 (MMP‐8) following root coverage procedures with either connective tissue graft or a collagen matrix, demonstrating that changes in MMP‐1 and MMP‐8 were associated with the final gain in keratinized tissue width and gingival thickness [66]. While the present study did not observe significant correlations between biomarkers expression and the final clinical outcomes, a statistically significant association between the levels of PDGF‐BB at 1 week and the patient‐reported time to recovery was found. Subjects with treated sites exhibiting a high expression of PDGF‐BB in the GCF at 1 week reported shorter times to recovery. It is also important to highlight that test sites demonstrated a statistically significant higher expression of ANG, IL‐1β, IL‐6, PDGF‐BB, and VEGF compared to untreated sites, while control sites displayed a statistically significantly greater expression of IL‐6 when compared to untreated sites. These results further indicate that the healing pattern of CCMX + rhPDGF occurred differently compared to that of CCMX without the growth factor. ANG, PDGF‐BB, and VEGF are known for being potent angiogenic biomarkers [47, 49, 69], and their increased levels in the GCF in the test group is probably related to the release of the growth factor from the matrix during early healing. The increased levels of the IL‐6 found in both groups compared to untreated sites may be related to the properties of this cytokine, which has been shown to promote a reparative environment [70]. These findings from the biomarker analysis align with the effects of rhPDGF in promoting angiogenesis and accelerating wound healing. This enhancement may facilitate quicker graft revascularization and resolution of the inflammatory phase, potentially resulting in less soft tissue shrinkage [18, 20, 21]. Future studies assessing the levels of wound healing biomarkers following root coverage procedures exploring their correlations with clinical outcomes and PROMs are encouraged.

Among the limitations of the present study, the relatively short follow‐up is to be mentioned. In addition, it should be highlighted that this study was designed as a secondary analysis of a randomized clinical trial that was powered on mRC. Therefore, when interpreting the findings of the present study, readers should bear in mind that it is possible that a lack of statistically significant difference between the two groups in terms of tissue perfusion outcomes could have been due to the limited sample size. Lastly, it would have been beneficial to study tissue perfusion of additional treatments and modalities for a thorough comparison, such as the use of a different control group (e.g., CCMX alone, not soaked with saline, or autogenous connective tissue graft), surgical approach, graft material, and/or biologic agent to the provided additional insights on HFUS and tissue perfusion assessment.

## 5 Conclusions

This investigation described the application of Doppler HFUS and GCF biomarker evaluation for characterizing tissue perfusion and wound healing at teeth exhibiting MAGRs and treated with CAF + CCMX, loaded either with saline or rhPDGF. Significant differences between the two groups were observed at the 2‐week time point, when DTPMs were calculated at the graft and flap ROI, independently. Tissue perfusion at the level of the CCMX was found to be significantly higher, in terms of pRI, pA, FI_mean_, and FI_tot_, when the matrix was loaded with rhPDGF. DTPMs assessed at 2 weeks at the level of the graft and the flap were correlated with patient‐reported time to recovery and the final clinical outcomes (mRC, CRC, Vol gain and GT gain). Different expressions of IL‐1β, PDFG‐BB, and VEGF were observed over 3 months at the test and control groups, with the 1‐week levels of PDGF‐BB that were associated with time to recovery.

## Conflicts of Interest

Drs. Lorenzo Tavelli and Shayan Barootchi have provided lectures sponsored by Geistlich Pharma AG, Wolhusen, Switzerland, and Lynch Biologics, Franklin, TN, USA. The other authors report no conflict of interest related to this study.

## Supporting information


Appendix S1

## Data Availability

The data that support the findings of this study are available from the corresponding author upon reasonable request.
