# Supplementary material for: Tissue Perfusion and Biomarkers Assessment Following Root Coverage Procedures
Source: J Periodontal Res. 2025 Jan 22;60(11):1117–31. doi: 10.1111/jre.13374 (PMC12779183; doi:10.1111/jre.13374)
Supplement: Supplementary file 1 — Appendix S1 [file JRE-60-1117-s001.docx]

**Tissue perfusion and biomarkers assessment following root coverage procedures**

Lorenzo Tavelli, Tu Nguyen, Maria Vera Rodriguez, Leonardo Mancini, William V. Giannobile,

and Shayan Barootchi

**Supplementary Appendix**

**Surgical intervention**

After local anesthesia, a split-full-split thickness flap was performed with a mini-blade (Mini Blade #67, Salvin Dental Specialties, Charlotte, USA) and a microsurgical periosteal elevator (Hu-Friedy, Chicago, USA) in a way that the interdental papillae were prepared split thickness, while the soft tissue in the mid-buccal portion was elevated full-thickness in the apical direction until exposing approximately 2 mm of buccal bone. The elevation was then continued split thickness using a 15c blade. The flap was released with a deep and a superficial incision, as previously described (de Sanctis and Zucchelli, 2007), until the flap was not able to reach a position approximately 2 mm coronal to the cemento-enamel junction, without residual tension. The anatomical papillae were de-epithelialized using a mini blade (Mini Blade #67, Salvin Dental Specialties, Charlotte, USA) or microsurgical scissors (Hu-Friedy, Chicago, USA). The root surfaces that were previously exposed to the oral cavity were scaled, planed and detoxified using 24% of EDTA for 2 minutes (Barootchi et al., 2018). For both groups, the xenogeneic cross-linked collagen matrix (CCM) was before extraorally trimmed with a 15c blade, in order to obtain a graft 3-4 mm in thickness and 8 mm in height. The width of the matrix was determined based on the characteristics of the recession defects. The XCM was then saturated with micro-injection of 1.5 cc of the solution contained in the randomization envelope. The graft was left in the dappen dish for 15 minutes as previously recommended (Rubins et al., 2013, Rubins et al., 2014). The solution was also applied on the dried root surfaces before stabilizing the graft. Simple interrupted sutures (6/0 and 7/0 PGA, AD Surgical, Sunnyvale, USA) engaging the graft and the de-epithelialized anatomical papillae were performed to stabilizing the XCM at the recipient bed, approximately 1 mm apical to the CEJ covering the root surface. Further stabilization of the graft was also achieved, if necessary, with additional mattress sutures (6/0 and 7/0 PGA, AD Surgical, Sunnyvale, USA) apical to the XCM, engaging the periosteum. The flap was then coronally advanced and stabilized approximately 2 mm coronal to the cemento-enamel junction using multiple sling sutures at the level of the papillae (6/0 and/or 7/0 polypropylene [Ethicon, Johnson & Johnson, Somerville, USA] or [AD Surgical, Sunnyvale, USA)]), completely covering the XCM. Simple interrupted sutures were performed at the level of the vertical releasing incisions, if any (7/0 polypropylene [Ethicon, Johnson & Johnson, Somerville, USA]).

**Post-operative regimen**

Patients were prescribed Amoxicillin (500 mg 3 times a day for 7 days), Ibuprofen (600 mg every 4-6 hours for the first 3 days, followed by its prescription as needed) and Chlorhexidine mouth rinse (0.12% twice daily for one minute for 14 days). The sutures were removed two weeks after the surgical procedure. Patients were instructed to resume mechanical tooth brushing at the operated area using an extra-soft bristle toothbrush.

**Expression of wound healing biomarkers over 3 months**

**Angiogenin (ANG).** The linear longitudinal regression using GEE model with untreated sites as the reference category demonstrated a statistically significant different pattern of ANG expression over the first 3 months between the test group and untreated sites (estimated coefficient of -20.3 (95% CI [-36.2, -4.4], p=0.012), with the peak of ANG observed at 1 week in the test group. Although a peak of ANG was also observed in the control group, the longitudinal regression did not reveal statistically significant differences between the control sites and untreated sites over 3 months in terms of ANG expression (estimated coefficient of -12.1 (95% CI [-26.6, 2.4], p=0.102). When the control group was used in the regression model as the reference, no significant differences were found between the test and control groups in terms of overall ANG expression over 3 months (estimated coefficient of -8.2 (95% CI [-29.0, 12.5], p=0.437). Pairwise comparisons at the individual time points exhibit a statistically significant difference of ANG values between control sites and untreated sites at 1 week (p<0.001), and between test sites and untreated sites at 1 week (p<0.001). The pairwise comparisons between the groups at other time points were not statistically significant.

**Basic fibroblast growth factor (bFGF).** No significant differences were observed between test sites and untreated sites (estimated coefficient of -0.021 (95% CI [-0.051, 0.009], p=0.164), nor between control sites and untreated sites (estimated coefficient of 0.007 (95% CI [-0.026, 0.041], p=0.670) in terms of expression of bFGF over 3 months. Similarly, there were no differences within the longitudinal pattern of bFGF between test and control groups (estimated coefficient of -0.029 (95% CI [-0.073, 0.016], p=0.210).

**Interleukin 1 beta (IL-1β).** A significant difference was found between the test and untreated sites in terms of the expression of IL-1β over 3 months (estimated coefficient of -1.13 (95% CI [-1.93, -0.34], p=0.005), while there was not a significant difference between control and untreated sites (estimated coefficient of -0.15 (95% CI [-0.73, 0.42], p=0.606). The longitudinal expression of IL-1β was significantly different between test and control sites (estimated coefficient of -0.98 (95% CI [-1.94, -0.03], p=0.043).

**Interleukin 6 (IL-6).** The regression model showed a statistically significant different longitudinal expression of IL-6 between test and untreated sites (estimated coefficient of -15.0 (95% CI [-26.0, -4.07], p=0.007), and between the control and untreated sites (estimated coefficient of -4.69 (95% CI [-8.46, -0.93], p=0.014). The expression of IL-6 over time at the test and control sites was not significantly different (estimated coefficient of -10.3 (95% CI [-21.9, 1.25], p=0.081).

**Interleukin 10 (IL-10).** No differences were found in the longitudinal expression of IL-10 between test and untreated sites (estimated coefficient of -0.03 (95% CI [-0.07, 0.00], p=0.099) and between control and untreated sites (estimated coefficient of -0.01 (95% CI [-0.04, 0.01], p=0.214). Similarly, there were no significant differences between test and control groups (estimated coefficient of -0.01 (95% CI [-0.04, 0.01], p=0.341).

**Platelet-derived growth factor-BB (PDGF-BB).** The regression model demonstrated a significant different pattern of PDGF-BB expression over 3 months between test sites and untreated sites (estimated coefficient of -0.47 (95% CI [-0.76, -0.18], p=0.02), while significant differences were not observed between control and untreated sites (estimated coefficient of -0.02 (95% CI [-0.10, 0.05], p=0.522). The pattern of expression of PDGF-BB between the test and control group was statistically significantly different (estimated coefficient of -0.44 (95% CI [-0.74, -0.14], p=0.003).

**Transforming growth factor beta-1 (TGF β1).** No significant differences were revealed by the longitudinal model assessing the expression of TGF β-1 between test and untreated sites (estimated coefficient of -542.8 (95% CI [-1573.2, 487.4], p=0.302), not between control and untreated sites (estimated coefficient of -476.0 (95% CI [-1171.7, 219.6], p=0.180). Similarly, no differences were observed between the test and control groups (estimated coefficient of -66.8 (95% CI [-1057.8, 924.2], p=0.895).

**Tissue inhibitor of metalloproteinases-2 (TIMP-2).** The longitudinal expression of TIMP-2 over 3 months was not statistically different between test and untreated sites (estimated coefficient of -11.4 (95% CI [-34.5, 11.6], p=0.331), nor between control and untreated sites (estimated coefficient of -12.8 (95% CI [-33.5, 7.77], p=0.222). Similarly, the longitudinal regression analysis did not reveal significant differences between test and control sites (estimated coefficient of 1.41 (95% CI [-24.6, 27.4], p=0.916). The increment of TIMP-2 in the test sites from baseline to 1 week was found to be statistically significant (p<0.001).

**Tumor necrosis factor-alpha (TNF-α).** No significant differences were found in the expression of TNF-α between test and untreated sites (estimated coefficient of -81.7 (95% CI [-167.3, 3.84], p=0.061). No significant differences were observed between control and untreated sites (estimated coefficient of -46.9 (95% CI [-114.9, 20.9], p=0.175). Similarly, there were no significant differences between test and control groups in terms of expression of TNF-α over 3 months (estimated coefficient of -34.7 (95% CI [-113.3, 43.7], p=0.386).

**Vascular endothelial growth factor (VEGF).** A statistically significant different expression of VEGF over 3 months was found between test and untreated sites (estimated coefficient of -4.71 (95% CI [-7.40, -2.03], p=0.001), while there was not statistically significant difference between the control and untreated sites (estimated coefficient of -0.67 (95% CI [-2.30, 0.947], p=0.413). The expression of VEGF over time was significantly different within the test and control groups (estimated coefficient of -4.04 (95% CI [-7.12, -0.95], p=0.010).

**Supplementary Table 1**. Intraoperative measurements of the cross-linked xenogeneic collagen matrix (CCMX) within the two groups. No significant differences were observed between the two groups.

| **Graft dimension** | **CCMX + saline** | **CCMX + rhPDGF** |
| --- | --- | --- |
| Length  (mean ± SD) (mm) | 28.75 ± 9.22 | 28.62 ± 6.19 |
| Height  (mean ± SD) (mm) | 8.21 ± 1.16 | 8.26 ± 1.52 |
| Thickness  (mean ± SD) (mm) | 3.52 ± 0.56 | 3.42 ± 0.46 |

**­Legend**. CCMX: cross-linked xenogeneic collagen matrix; rhPDGF: recombinant human platelet-derived growth factor-BB; SD: standard deviation.

**Supplementary Table 2.** Correlations between the biomarker expression at different time point using the Spearman’s correlation test. Note that only correlations with coefficient (R) ≥ 0.7 were reported.

|  | **Control sites**  (15 Subjects, 44 sites) | | | **Test sites**  (15 Subjects, 47 sites) | | |
| --- | --- | --- | --- | --- | --- | --- |
| Time (days) | Correlations | R | p-value | Correlation | R | p-value |
| 0 | TIMP-2 and ANG | 0.87 | <0.001 | IL-10 and bFGF | 0.83 | <0.001 |
|  |  |  |  | PDGF-BB and bFGF | 0.78 | 0.001 |
|  |  |  |  | PDGF-BB and IL-10 | 0.74 | 0.001 |
|  |  |  |  | TNF-α and bFGF | 0.74 | 0.002 |
| 7 | IL-6 and ANG | 0.75 | 0.002 | IL-6 and ANG | 0.72 | 0.002 |
|  |  |  |  | PDGF-BB and ANG | 0.71 | 0.003 |
|  |  |  |  | TIMP-2 and ANG | 0.75 | 0.001 |
|  | TIMP-2 and ANG | 0.88 | <0.001 | TIMP-2 and bFGF | 0.71 | 0.003 |
|  |  |  |  | TIMP-2 and IL-6 | 0.84 | <0.001 |
|  | TNF-α and TGF β1 | 0.84 | <0.001 | TIMP-2 and PDGF-BB | 0.9 | <0.001 |
|  |  |  |  | TNF-α and TIMP-2 | 0.75 | 0.001 |
|  | VEGF and ANG | 0.85 | <0.001 | VEGF and ANG | 0.71 | 0.003 |
|  |  |  |  | VEGF and IL-6 | 0.88 | <0.001 |
|  | VEGF and TIMP-2 | 0.88 | <0.001 | VEGF and PDGF-BB | 0.86 | <0.001 |
|  |  |  |  | VEGF and TIMP-2 | 0.88 | <0.001 |
| 14 | IL-10 and ANG | 0.81 | <0.001 | PDGF-BB and bFGF | 0.88 | <0.001 |
|  | PDGF-BB and IL-6 | 0.79 | 0.001 | TIMP-2 and ANG | 0.86 | <0.001 |
|  | TGFb1 and bFGF | 0.7 | 0.017 | TIMP-2 and IL-1β | 0.79 | 0.001 |
|  | TGFb1 and IL6 | 0.77 | 0.001 | VEGF and IL-1β | 0.78 | 0.001 |
|  | TGFb1 and PDGF-BB | 0.95 | <0.001 |  |  |  |
|  | TIMP-2 and ANG | 0.7 | 0.005 | VEGF and TIMP-2 | 0.78 | 0.001 |
|  | TNF-α and TGF-β1 | 0.7 | 0.006 |  |  |  |
| 30 | TGF β1 and IL-6 | 0.78 | 0.001 | IL-1β and bFGF | 0.73 | 0.003 |
|  | TIMP2 and ANG | 0.71 | 0.005 | IL-6 and IL1b | 0.75 | 0.002 |
|  | TNF-α and IL-6 | 0.8 | 0.001 | PDGF-BB and bFGF | 0.78 | 0.001 |
|  |  |  |  | PDGF-BB and IL-1β | 0.73 | 0.003 |
|  | TNF-α and TGF β1 | 0.92 | <0.001 | PDGF-BB and IL-6 | 0.81 | <0.001 |
|  |  |  |  | TGF β1 and IL-6 | 0.75 | 0.002 |
|  | VEGF and ANG | 0.82 | <0.001 | TIMP-2 and ANG | 0.73 | 0.003 |
|  |  |  |  | TNF-α and IL-6 | 0.74 | 0.002 |
|  | VEGF and TIMP2 | 0.8 | 0.001 | TNF-α and PDGF-BB | 0.71 | 0.004 |
|  |  |  |  | VEGF and PDGF-BB | 0.75 | 0.002 |
| 90 | PDGF-BB and IL-6 | 0.79 | 0.001 | PDGF-BB and bFGF | 0.71 | 0.005 |
|  | TGF β1 and bFGF | 0.95 | <0.001 | TGF β1 and IL6 | 0.7 | 0.007 |
|  | TNF-α and TIMP2 | 0.74 | 0.003 | TIMP-2 and ANG | 0.72 | 0.004 |
|  | VEGF and IL-1β | 0.85 | <0.001 |  |  |  |
|  | VEGF and TIMP-2 | 0.67 | 0.008 | VEGF and TNF-α | 0.84 | <0.001 |
|  | VEGF and TNF-α | 0.77 | 0.001 |  |  |  |

**Supplementary Figure 1.** CONSORT flowchart

**
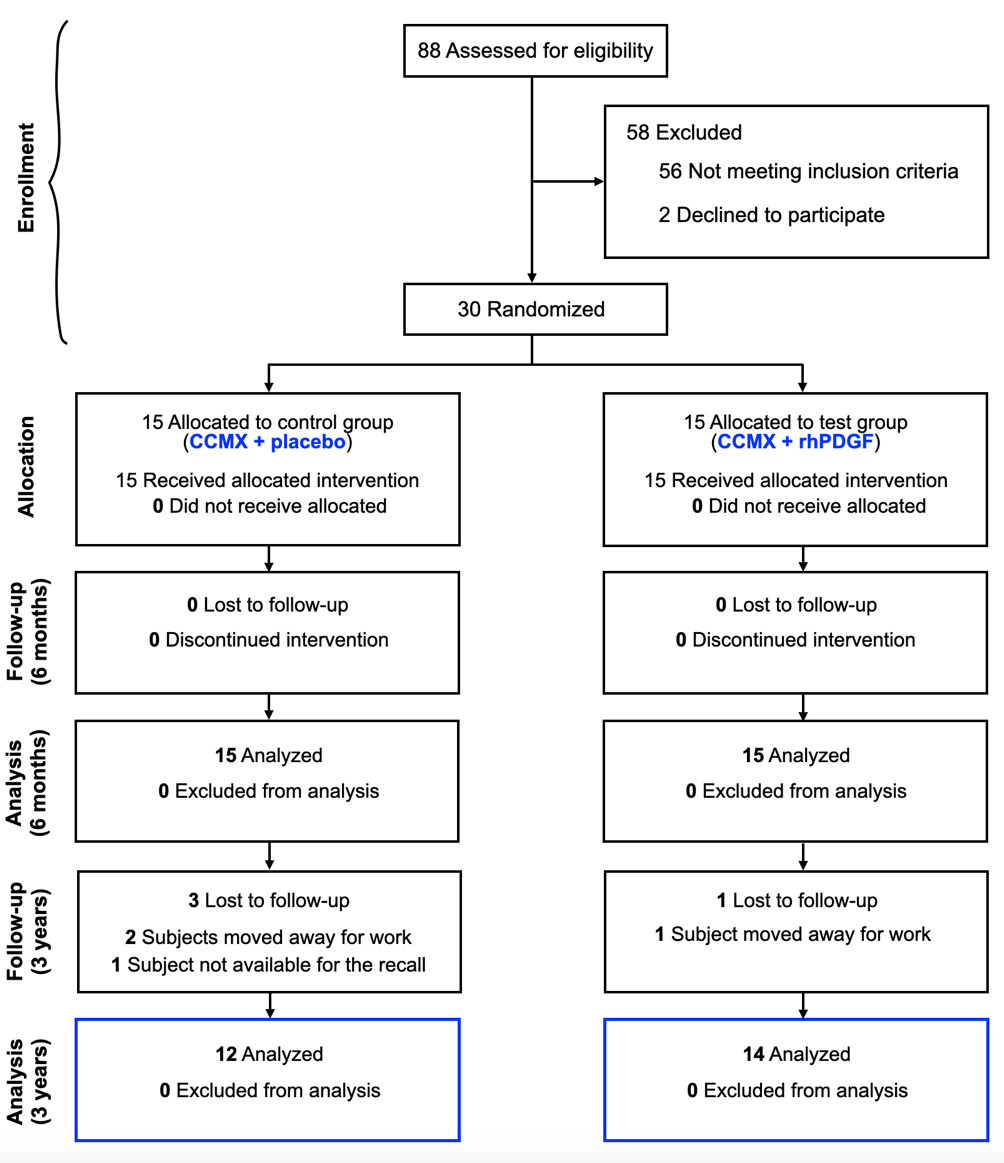
**

**Supplementary Figure 2**. Graphs depicting the tissue perfusion changes over 6 months within the soft tissue region of interests at the midfacial aspect of the treated teeth.


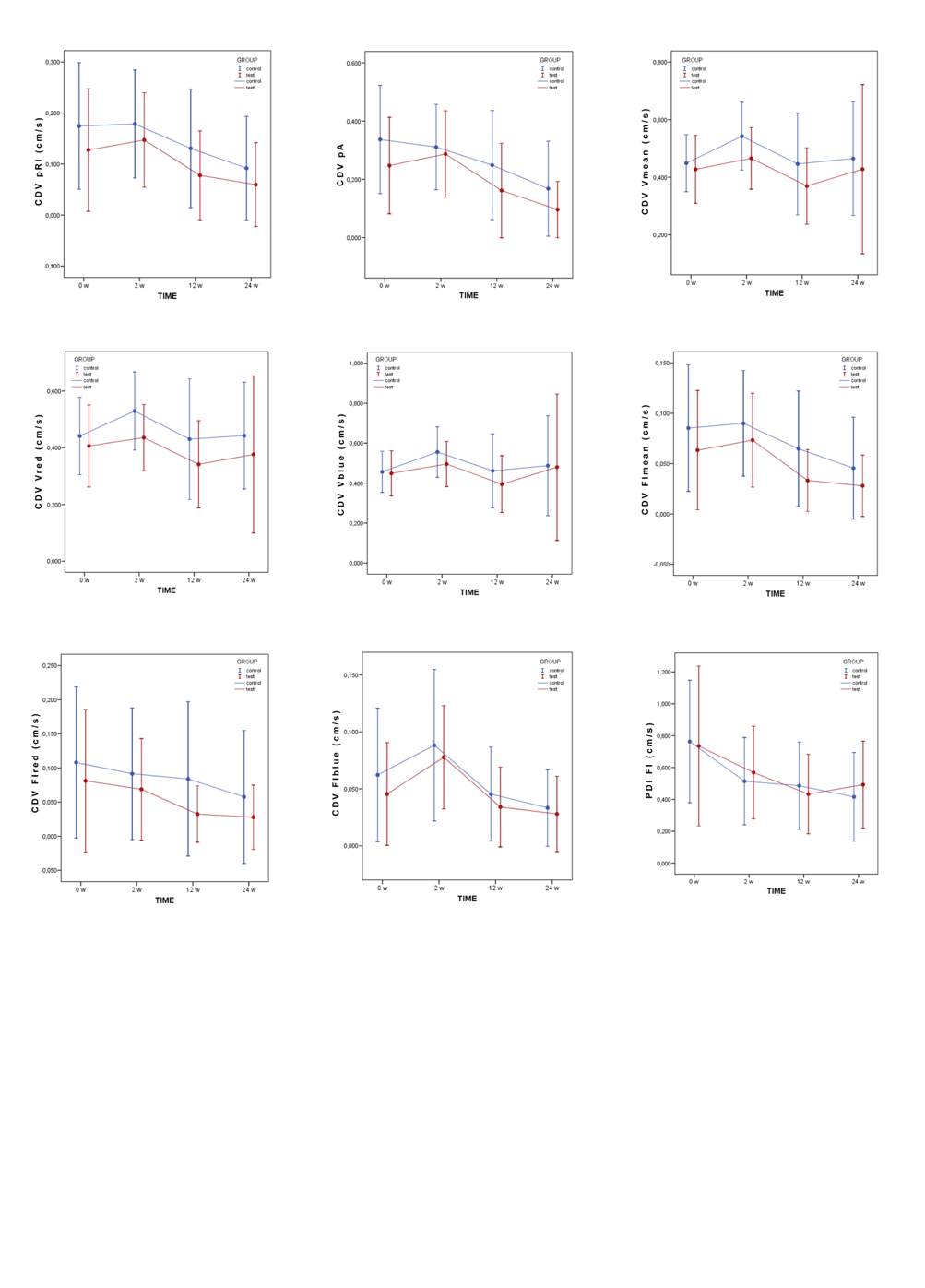


**Supplementary Figure 3**. Graphs depicting the tissue perfusion changes over 6 months within the soft tissue region of interests at the interproximal aspect of the treated teeth.


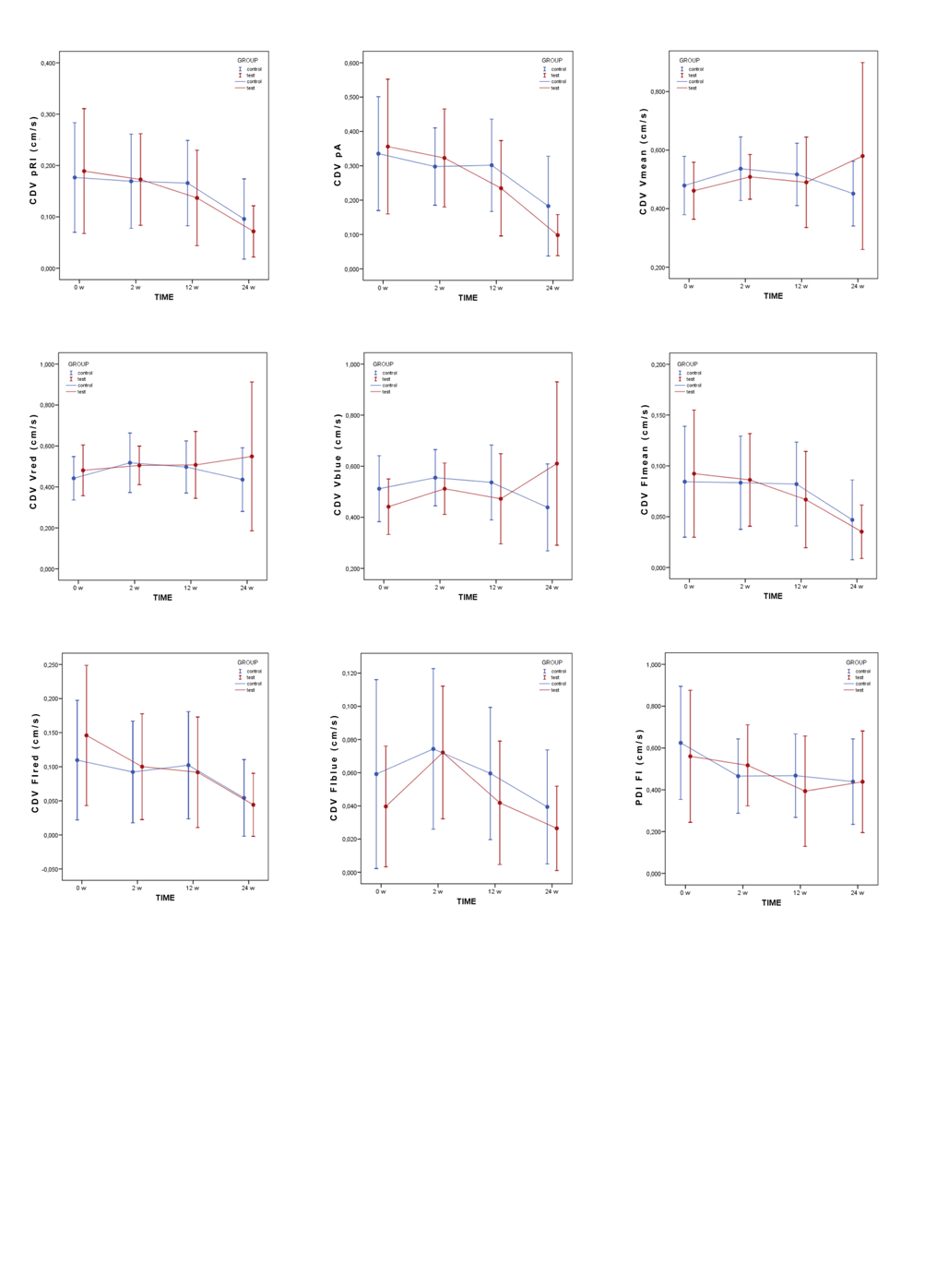


**Supplementary Figure 4**. Graphs depicting the tissue perfusion changes over 6 months within the soft tissue region of interests at the transverse aspect of the treated teeth.


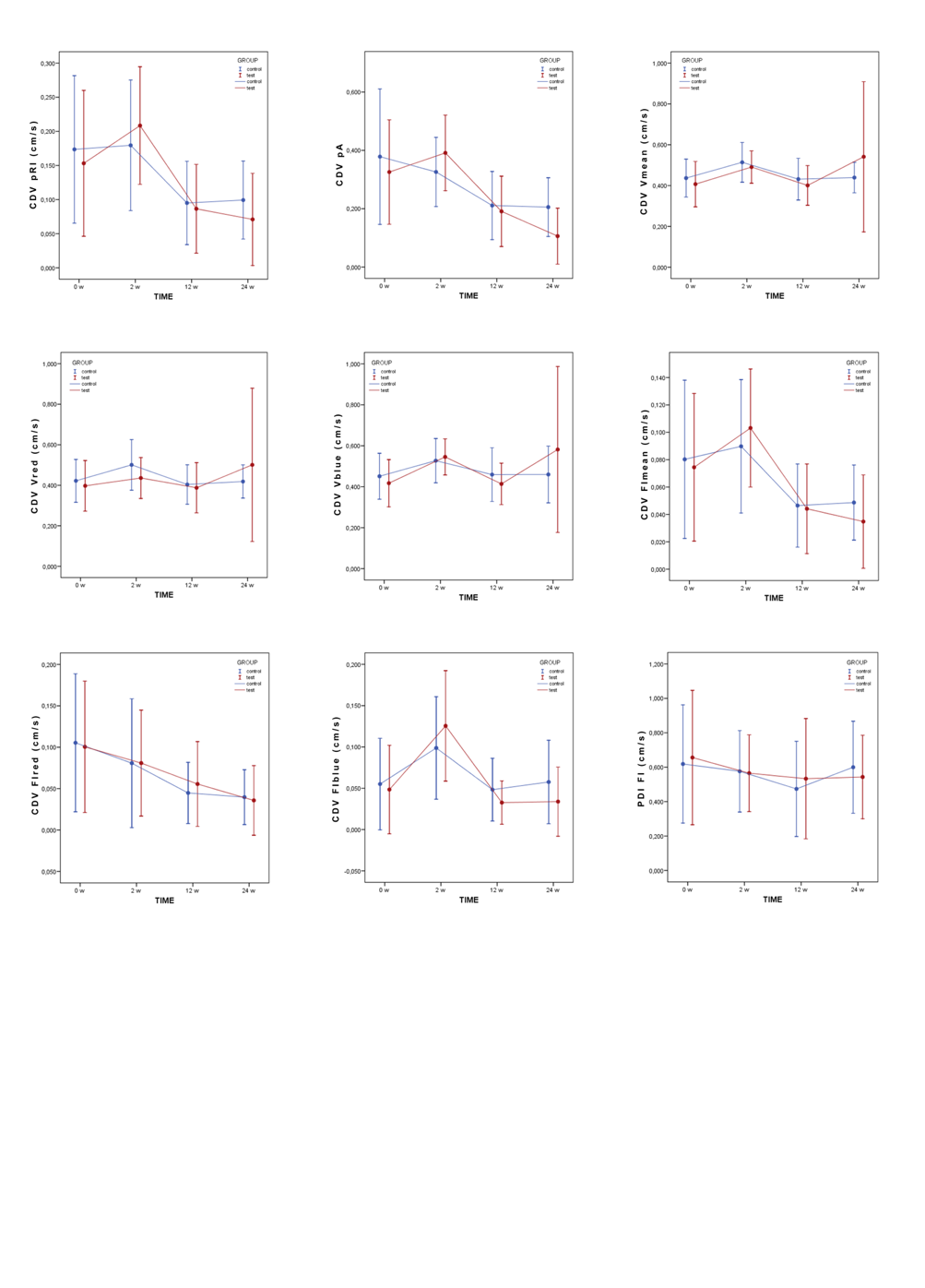


**Supplementary Figure 5**. Graphs depicting the expression of the different biomarkers over 3 months at the treated (test and control group) and at the untreated sites.


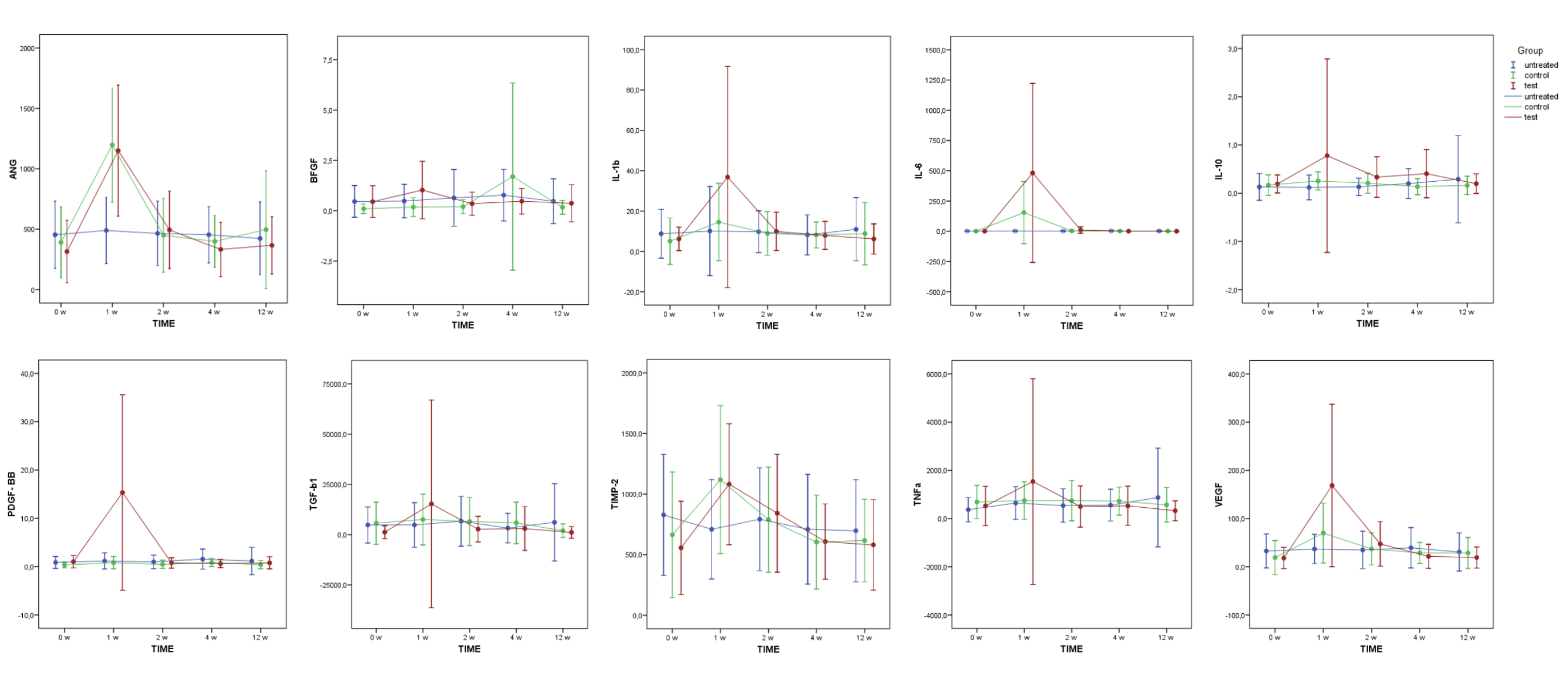


**Supplementary Figure 6**. Scatterplot graphs depicting the slope of the different biomarkers at the treated (test and control group) and at the untreated sites based on the results of the regression analyses.


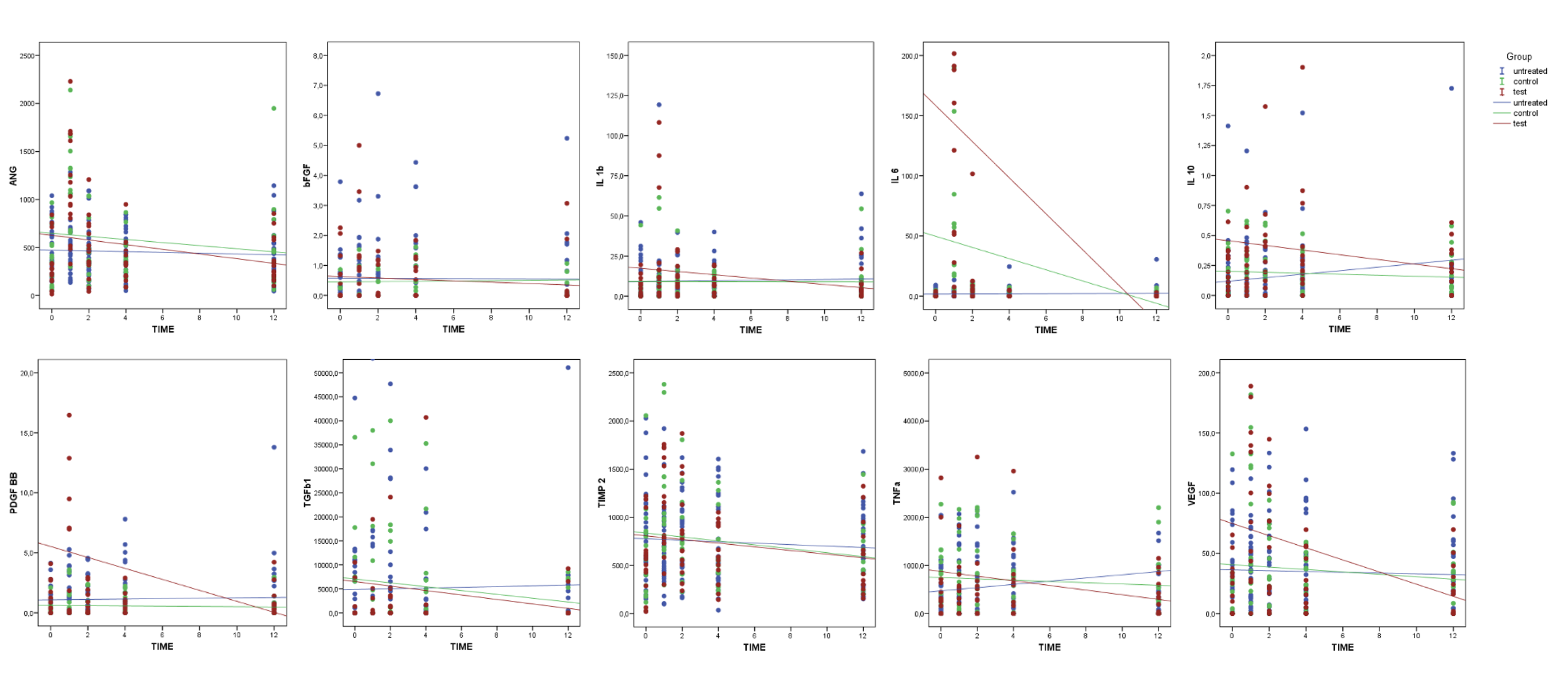


**Regression analyses**

Longitudinal regression analysis assessing **CDV pRI at the midfacial aspect** using GEE model and the control group as reference category.

|  | **B** | **SE** | **95% Wald Confidence Interval** | |  |
| --- | --- | --- | --- | --- | --- |
|  |  |  | **Lower** | **Upper** | **p-value** |
| **Intercept** | 0.179 | 0.0144 | 0.150 | 0.207 | **<0.001***** |
| **Group=Test** | -0.042 | 0.0199 | -0.081 | -0.003 | **0.035*** |
| **Group=Control (ref.)** | 0 | . | . | . | . |
| **Time** | -0.004 | 0.0006 | -0.005 | -0.002 | **<0.001***** |
| **Group=Test*Time** | 0.000 | 0.0012 | -0.002 | 0.002 | 0.884 |
| **Group=Control*Time** | 0 | . | . | . | . |
| **(Scale)** | 0.011 |  |  |  |  |

*p<0.05; **p<0.01; ***p<0.001

Longitudinal regression analysis assessing **CDV pA at the midfacial aspect** using GEE model and the control group as reference category.

|  | **B** | **SE** | **95% Wald Confidence Interval** | |  |
| --- | --- | --- | --- | --- | --- |
|  |  |  | **Lower** | **Upper** | **p-value** |
| **Intercept** | 0.331 | 0.0225 | 0.287 | 0.375 | **<0.001***** |
| **Group=Test** | -0.061 | 0.0321 | -0.124 | 0.002 | 0.058 |
| **Group=Control (ref.)** | 0 | . | . | . | . |
| **Time** | -0.007 | 0.0011 | -0.009 | -0.005 | **<0.001***** |
| **Group=Test*Time** | -0.001 | 0.0019 | -0.004 | 0.003 | 0.710 |
| **Group=Control*Time** | 0 | . | . | . | . |
| **(Scale)** | 0.025 |  |  |  |  |

*p<0.05; **p<0.01; ***p<0.001

Longitudinal regression analysis assessing **CDV Vmean at the midfacial aspect** using GEE model and the control group as reference category.

|  | **B** | **SE** | **95% Wald Confidence Interval** | |  |
| --- | --- | --- | --- | --- | --- |
|  |  |  | **Lower** | **Upper** | **p-value** |
| **Intercept** | 0.485 | 0.0150 | 0.455 | 0.514 | **<0.001***** |
| **Group=Test** | -0.050 | 0.0217 | -0.092 | -0.007 | **0.022*** |
| **Group=Control (ref.)** | 0 | . | . | . | . |
| **Time** | -0.001 | 0.0014 | -0.004 | 0.002 | 0.406 |
| **Group=Test*Time** | 0.001 | 0.0021 | -0.004 | 0.004 | 0.971 |
| **Group=Control*Time** | 0 | . | . | . | . |
| **(Scale)** | 0.029 |  |  |  |  |

*p<0.05; **p<0.01; ***p<0.001

Longitudinal regression analysis assessing **CDV Vred at the midfacial aspect** using GEE model and the control group as reference category.

|  | **B** | **SE** | **95% Wald Confidence Interval** | |  |
| --- | --- | --- | --- | --- | --- |
|  |  |  | **Lower** | **Upper** | **p-value** |
| **Intercept** | 0.475 | 0.0158 | 0.444 | 0.506 | **<0.001***** |
| **Group=Test** | -0.063 | 0.0232 | -0.108 | -0.017 | **0.007**** |
| **Group=Control (ref.)** | 0 | . | . | . | . |
| **Time** | -0.002 | 0.0013 | -0.004 | 0.001 | 0.195 |
| **Group=Test*Time** | -0.001 | 0.0021 | -0.005 | 0.003 | 0.767 |
| **Group=Control*Time** | 0 | . | . | . | . |
| **(Scale)** | 0.032 |  |  |  |  |

*p<0.05; **p<0.01; ***p<0.001

Longitudinal regression analysis assessing **CDV V_blue_ at the midfacial aspect** using GEE model and the control group as reference category.

|  | **B** | **SE** | **95% Wald Confidence Interval** | |  |
| --- | --- | --- | --- | --- | --- |
|  |  |  | **Lower** | **Upper** | **p-value** |
| **Intercept** | 0.494 | 0.0190 | 0.457 | 0.531 | **<0.001***** |
| **Group=Test** | -0.037 | 0.0250 | -0.086 | 0.012 | 0.143 |
| **Group=Control (ref.)** | 0 | . | . | . | . |
| **Time** | -0.001 | 0.0018 | -0.004 | 0.003 | 0.726 |
| **Group=Test*Time** | 0.000 | 0.0026 | -0.005 | 0.005 | 0.863 |
| **Group=Control*Time** | 0 | . | . | . | . |
| **(Scale)** | 0.040 |  |  |  |  |

*p<0.05; **p<0.01; ***p<0.001

Longitudinal regression analysis assessing **CDV FI_mean_ at the midfacial aspect** using GEE model and the control group as reference category.

|  | **B** | **SE** | **95% Wald Confidence Interval** | |  |
| --- | --- | --- | --- | --- | --- |
|  |  |  | **Lower** | **Upper** | **p-value** |
| **Intercept** | 0.088 | 0.0080 | 0.073 | 0.104 | **<0.001***** |
| **Group=Test** | -0.021 | 0.0104 | -0.042 | -0.001 | **0.041*** |
| **Group=Control (ref.)** | 0 | . | . | . | **.** |
| **Time** | -0.002 | 0.0003 | -0.002 | -0.001 | **<0.001***** |
| **Group=Test*Time** | 0.005 | 0.0005 | -0.001 | 0.001 | 0.954 |
| **Group=Control*Time** | 0 | . | . | . | . |
| **(Scale)** | 0.002 |  |  |  |  |

*p<0.05; **p<0.01; ***p<0.001

Longitudinal regression analysis assessing **CDV FI_red_ at the midfacial aspect** using GEE model and the control group as reference category.

|  | **B** | **SE** | **95% Wald Confidence Interval** | |  |
| --- | --- | --- | --- | --- | --- |
|  |  |  | **Lower** | **Upper** | **p-value** |
| **Intercept** | 0.103 | 0.0181 | 0.068 | 0.139 | **<0.001***** |
| **Group=Test** | -0.030 | 0.0205 | -0.070 | 0.010 | 0.146 |
| **Group=Control (ref.)** | 0 | . | . | . | . |
| **Time** | -0.002 | 0.0004 | -0.003 | -0.001 | **<0.001***** |
| **Group=Test*Time** | 0.000 | 0.0007 | -0.002 | 0.001 | 0.634 |
| **Group=Control*Time** | 0 | . | . | . | . |
| **(Scale)** | 0.008 |  |  |  |  |

*p<0.05; **p<0.01; ***p<0.001

Longitudinal regression analysis assessing **CDV FI_blue_ at the midfacial aspect** using GEE model and the control group as reference category.

|  | **B** | **SE** | **95% Wald Confidence Interval** | |  |
| --- | --- | --- | --- | --- | --- |
|  |  |  | **Lower** | **Upper** | **p-value** |
| **Intercept** | 0.073 | 0.0096 | 0.055 | 0.092 | **<0.001***** |
| **Group=Test** | -0.012 | 0.0111 | -0.034 | 0.009 | 0.263 |
| **Group=Control (ref.)** | 0 | . | . | . | . |
| **Time** | -0.002 | 0.0003 | -0.002 | -0.001 | **<0.001***** |
| **Group=Test*Time** | 0.000 | 0.0005 | -0.001 | 0.001 | 0.594 |
| **Group=Control*Time** | 0 | . | . | . | . |
| **(Scale)** | 0.002 |  |  |  |  |

*p<0.05; **p<0.01; ***p<0.001

Longitudinal regression analysis assessing **PDI FI at the midfacial aspect** using GEE model and the control group as reference category.

|  | **B** | **SE** | **95% Wald Confidence Interval** | |  |
| --- | --- | --- | --- | --- | --- |
|  |  |  | **Lower** | **Upper** | **p-value** |
| **Intercept** | 0.653 | 0.0361 | 0.582 | 0.724 | **<0.001***** |
| **Group=Test** | -0.023 | 0.0706 | -0.161 | 0.116 | 0.749 |
| **Group=Control (ref.)** | 0 | . | . | . | . |
| **Time** | -0.011 | 0.0016 | -0.014 | -0.008 | **<0.001***** |
| **Group=Test*Time** | 0.003 | 0.0041 | -0.005 | 0.011 | 0.466 |
| **Group=Control*Time** | 0 | . | . | . | . |
| **(Scale)** | 0.111 |  |  |  |  |

*p<0.05; **p<0.01; ***p<0.001

Longitudinal regression analysis assessing **CDV pRI at the interproximal aspect** using GEE model and the control group as reference category.

|  | **B** | **SE** | **95% Wald Confidence Interval** | |  |
| --- | --- | --- | --- | --- | --- |
|  |  |  | **Lower** | **Upper** | **p-value** |
| **Intercept** | 0.182 | 0.0110 | 0.160 | 0.203 | **<0.001***** |
| **Group=Test** | 0.005 | 0.0208 | -0.035 | 0.046 | 0.796 |
| **Group=Control (ref.)** | 0 | . | . | . | . |
| **Time** | -0.003 | 0.0008 | -0.005 | -0.002 | **<0.001***** |
| **Group=Test*Time** | -0.002 | 0.0013 | -0.004 | 0.001 | 0.224 |
| **Group=Control*Time** | 0 | . | . | . | . |
| **(Scale)** | 0.008 |  |  |  |  |

*p<0.05; **p<0.01; ***p<0.001

Longitudinal regression analysis assessing **CDV pA at the interproximal aspect** using GEE model and the control group as reference category.

|  | **B** | **SE** | **95% Wald Confidence Interval** | |  |
| --- | --- | --- | --- | --- | --- |
|  |  |  | **Lower** | **Upper** | **p-value** |
| **Intercept** | 0.332 | 0.0179 | 0.297 | 0.367 | **<0.001***** |
| **Group=Test** | 0.020 | 0.0343 | -0.048 | 0.087 | 0.568 |
| **Group=Control (ref.)** | 0 | . | . | . | . |
| **Time** | -0.005 | 0.0014 | -0.008 | -0.003 | **<0.001***** |
| **Group=Test*Time** | -0.005 | 0.0022 | -0.009 | -0.001 | **0.025*** |
| **Group=Control*Time** | 0 | . | . | . | . |
| **(Scale)** | 0.020 |  |  |  |  |

*p<0.05; **p<0.01; ***p<0.001

Longitudinal regression analysis assessing **CDV Vmean at the interproximal aspect** using GEE model and the control group as reference category.

|  | **B** | **SE** | **95% Wald Confidence Interval** | |  |
| --- | --- | --- | --- | --- | --- |
|  |  |  | **Lower** | **Upper** | **p-value** |
| **Intercept** | 0.515 | 0.0105 | 0.494 | 0.535 | **<0.001***** |
| **Group=Test** | -0.041 | 0.0181 | -0.077 | -0.006 | **0.022*** |
| **Group=Control (ref.)** | 0 | . | . | . | **.** |
| **Time** | -0.002 | 0.0009 | -0.004 | 0.000 | **0.036*** |
| **Group=Test*Time** | 0.006 | 0.0032 | -0.001 | 0.012 | 0.073 |
| **Group=Control*Time** | 0 | . | . | . | . |
| **(Scale)** | 0.022 |  |  |  |  |

*p<0.05; **p<0.01; ***p<0.001

Longitudinal regression analysis assessing **CDV Vred at the interproximal aspect** using GEE model and the control group as reference category.

|  | **B** | **SE** | **95% Wald Confidence Interval** | |  |
| --- | --- | --- | --- | --- | --- |
|  |  |  | **Lower** | **Upper** | **p-value** |
| **Intercept** | 0.486 | 0.0187 | 0.449 | 0.523 | **<0.001***** |
| **Group=Test** | 0.002 | 0.0240 | -0.045 | 0.049 | 0.928 |
| **Group=Control (ref.)** | 0 | . | . | . | . |
| **Time** | -0.001 | 0.0013 | -0.004 | 0.001 | 0.346 |
| **Group=Test*Time** | 0.004 | 0.0037 | -0.004 | 0.011 | 0.330 |
| **Group=Control*Time** | 0 | . | . | . | . |
| **(Scale)** | 0.030 |  |  |  |  |

*p<0.05; **p<0.01; ***p<0.001

Longitudinal regression analysis assessing **CDV V_blue_ at the interproximal aspect** using GEE model and the control group as reference category.

|  | **B** | **SE** | **95% Wald Confidence Interval** | |  |
| --- | --- | --- | --- | --- | --- |
|  |  |  | **Lower** | **Upper** | **p-value** |
| **Intercept** | 0.545 | 0.0138 | 0.518 | 0.572 | **<0.001***** |
| **Group=Test** | -0.087 | 0.0226 | -0.131 | -0.042 | **<0.001***** |
| **Group=Control (ref.)** | 0 | . | . | . | **.** |
| **Time** | -0.003 | 0.0015 | -0.006 | 0.000 | **0.024*** |
| **Group=Test*Time** | 0.009 | 0.0033 | 0.002 | 0.015 | **0.008**** |
| **Group=Control*Time** | 0 | . | . | . | . |
| **(Scale)** | 0.029 |  |  |  |  |

*p<0.05; **p<0.01; ***p<0.001

Longitudinal regression analysis assessing **CDV FI_mean_ at the interproximal aspect** using GEE model and the control group as reference category.

|  | **B** | **SE** | **95% Wald Confidence Interval** | |  |
| --- | --- | --- | --- | --- | --- |
|  |  |  | **Lower** | **Upper** | **p-value** |
| **Intercept** | 0.088 | 0.0056 | 0.077 | 0.099 | **<0.001***** |
| **Group=Test** | 0.004 | 0.0104 | -0.016 | 0.024 | 0.706 |
| **Group=Control (ref.)** | 0 | . | . | . | . |
| **Time** | -0.001 | 0.0004 | -0.002 | -0.001 | **<0.001***** |
| **Group=Test*Time** | -0.001 | 0.0007 | -0.002 | 0.000 | 0.189 |
| **Group=Control*Time** | 0 | . | . | . | . |
| **(Scale)** | 0.002 |  |  |  |  |

*p<0.05; **p<0.01; ***p<0.001

Longitudinal regression analysis assessing **CDV FI_red_ at the interproximal aspect** using GEE model and the control group as reference category.

|  | **B** | **SE** | **95% Wald Confidence Interval** | |  |
| --- | --- | --- | --- | --- | --- |
|  |  |  | **Lower** | **Upper** | **p-value** |
| **Intercept** | 0.108 | 0.0103 | 0.088 | 0.128 | **<0.001***** |
| **Group=Test** | 0.020 | 0.0175 | -0.014 | 0.054 | 0.252 |
| **Group=Control (ref.)** | 0 | . | . | . | . |
| **Time** | -0.002 | 0.0007 | -0.003 | 0.000 | **0.007**** |
| **Group=Test*Time** | -0.002 | 0.0010 | -0.004 | 0.000 | 0.127 |
| **Group=Control*Time** | 0 | . | . | . | . |
| **(Scale)** | 0.006 |  |  |  |  |

*p<0.05; **p<0.01; ***p<0.001

Longitudinal regression analysis assessing **CDV FI_blue_ at the interproximal aspect** using GEE model and the control group as reference category.

|  | **B** | **SE** | **95% Wald Confidence Interval** | |  |
| --- | --- | --- | --- | --- | --- |
|  |  |  | **Lower** | **Upper** | **p-value** |
| **Intercept** | 0.068 | 0.0069 | 0.055 | 0.082 | **<0.001***** |
| **Group=Test** | -0.011 | 0.0085 | -0.028 | 0.005 | 0.185 |
| **Group=Control (ref.)** | 0 | . | . | . | . |
| **Time** | -0.001 | 0.0004 | -0.002 | 0.000 | **0.011**** |
| **Group=Test*Time** | 0.000 | 0.0006 | -0.001 | 0.001 | 0.805 |
| **Group=Control*Time** | 0 | . | . | . | . |
| **(Scale)** | 0.002 |  |  |  |  |

*p<0.05; **p<0.01; ***p<0.001

Longitudinal regression analysis assessing **PDI FI at the interproximal aspect** using GEE model and the control group as reference category.

|  | **B** | **SE** | **95% Wald Confidence Interval** | |  |
| --- | --- | --- | --- | --- | --- |
|  |  |  | **Lower** | **Upper** | **p-value** |
| **Intercept** | 0.867 | 0.0215 | 0.825 | 0.909 | **<0.001***** |
| **Group=Test** | -0.037 | 0.0399 | -0.115 | 0.041 | 0.356 |
| **Group=Control (ref.)** | 0 | . | . | . | . |
| **Time** | -0.003 | 0.0016 | -0.006 | 0.000 | 0.078 |
| **Group=Test*Time** | 0.003 | 0.0026 | -0.002 | 0.008 | 0.235 |
| **Group=Control*Time** | 0 | . | . | . | . |
| **(Scale)** | 0.034 |  |  |  |  |

*p<0.05; **p<0.01; ***p<0.001

Longitudinal regression analysis assessing **CDV pRI at the transverse aspect** using GEE model and the control group as reference category.

|  | **B** | **SE** | **95% Wald Confidence Interval** | |  |
| --- | --- | --- | --- | --- | --- |
|  |  |  | **Lower** | **Upper** | **p-value** |
| **Intercept** | 0.172 | 0.0135 | 0.146 | 0.199 | **<0.001***** |
| **Group=Test** | 0.010 | 0.0204 | -0.030 | 0.050 | 0.638 |
| **Group=Control (ref.)** | 0 | . | . | . | . |
| **Time** | -0.004 | 0.0011 | -0.006 | -0.002 | **<0.001***** |
| **Group=Test*Time** | -0.001 | 0.0013 | -0.004 | 0.001 | 0.249 |
| **Group=Control*Time** | 0 | . | . | . | . |
| **(Scale)** | 0.008 |  |  |  |  |

*p<0.05; **p<0.01; ***p<0.001

Longitudinal regression analysis assessing **CDV pA at the transverse aspect** using GEE model and the control group as reference category.

|  | **B** | **SE** | **95% Wald Confidence Interval** | |  |
| --- | --- | --- | --- | --- | --- |
|  |  |  | **Lower** | **Upper** | **p-value** |
| **Intercept** | 0.349 | 0.0171 | 0.316 | 0.383 | **<0.001***** |
| **Group=Test** | 0.016 | 0.0327 | -0.048 | 0.080 | 0.615 |
| **Group=Control (ref.)** | 0 | . | . | . | . |
| **Time** | -0.008 | 0.0020 | -0.011 | -0.004 | **<0.001***** |
| **Group=Test*Time** | -0.004 | 0.0023 | -0.009 | 0.000 | 0.080 |
| **Group=Control*Time** | 0 | . | . | . | . |
| **(Scale)** | 0.023 |  |  |  |  |

*p<0.05; **p<0.01; ***p<0.001

Longitudinal regression analysis assessing **CDV Vmean at the transverse aspect** using GEE model and the control group as reference category.

|  | **B** | **SE** | **95% Wald Confidence Interval** | |  |
| --- | --- | --- | --- | --- | --- |
|  |  |  | **Lower** | **Upper** | **p-value** |
| **Intercept** | 0.469 | 0.0141 | 0.441 | 0.496 | **<0.001***** |
| **Group=Test** | -0.034 | 0.0194 | -0.072 | 0.004 | 0.080 |
| **Group=Control (ref.)** | 0 | . | . | . | . |
| **Time** | -0.002 | 0.0008 | -0.003 | 0.000 | **0.036*** |
| **Group=Test*Time** | 0.004 | 0.0031 | -0.002 | 0.010 | 0.191 |
| **Group=Control*Time** | 0 | . | . | . | . |
| **(Scale)** | 0.020 |  |  |  |  |

*p<0.05; **p<0.01; ***p<0.001

Longitudinal regression analysis assessing **CDV Vred at the transverse aspect** using GEE model and the control group as reference category.

|  | **B** | **SE** | **95% Wald Confidence Interval** | |  |
| --- | --- | --- | --- | --- | --- |
|  |  |  | **Lower** | **Upper** | **p-value** |
| **Intercept** | 0.453 | 0.0167 | 0.421 | 0.486 | **<0.001***** |
| **Group=Test** | -0.049 | 0.0246 | -0.097 | 0.000 | **0.048*** |
| **Group=Control (ref.)** | 0 | . | . | . | . |
| **Time** | -0.002 | 0.0011 | -0.004 | 0.000 | 0.072 |
| **Group=Test*Time** | 0.005 | 0.0032 | -0.002 | 0.011 | 0.153 |
| **Group=Control*Time** | 0 | . | . | . | . |
| **(Scale)** | 0.023 |  |  |  |  |

*p<0.05; **p<0.01; ***p<0.001

Longitudinal regression analysis assessing **CDV V_blue_ at the transverse aspect** using GEE model and the control group as reference category.

|  | **B** | **SE** | **95% Wald Confidence Interval** | |  |
| --- | --- | --- | --- | --- | --- |
|  |  |  | **Lower** | **Upper** | **p-value** |
| **Intercept** | 0.484 | 0.0159 | 0.453 | 0.515 | **<0.001***** |
| **Group=Test** | -0.019 | 0.0214 | -0.061 | 0.023 | 0.369 |
| **Group=Control (ref.)** | 0 | . | . | . | . |
| **Time** | -0.001 | 0.0011 | -0.003 | 0.001 | 0.316 |
| **Group=Test*Time** | 0.004 | 0.0037 | -0.004 | 0.011 | 0.322 |
| **Group=Control*Time** | 0 | . | . | . | . |
| **(Scale)** | 0.026 |  |  |  |  |

*p<0.05; **p<0.01; ***p<0.001

Longitudinal regression analysis assessing **CDV FI_mean_ at the transverse aspect** using GEE model and the control group as reference category.

|  | **B** | **SE** | **95% Wald Confidence Interval** | |  |
| --- | --- | --- | --- | --- | --- |
|  |  |  | **Lower** | **Upper** | **p-value** |
| **Intercept** | 0.083 | 0.0070 | 0.069 | 0.096 | **<0.001***** |
| **Group=Test** | 0.007 | 0.0102 | -0.013 | 0.027 | 0.500 |
| **Group=Control (ref.)** | 0 | . | . | . | . |
| **Time** | -0.002 | 0.0005 | -0.003 | -0.001 | **0.001**** |
| **Group=Test*Time** | -0.001 | 0.0006 | -0.002 | 0.000 | 0.208 |
| **Group=Control*Time** | 0 | . | . | . | . |
| **(Scale)** | 0.002 |  |  |  |  |

*p<0.05; **p<0.01; ***p<0.001

Longitudinal regression analysis assessing **CDV FI_red_ at the transverse aspect** using GEE model and the control group as reference category.

|  | **B** | **SE** | **95% Wald Confidence Interval** | |  |
| --- | --- | --- | --- | --- | --- |
|  |  |  | **Lower** | **Upper** | **p-value** |
| **Intercept** | 0.093 | 0.0117 | 0.070 | 0.116 | **<0.001***** |
| **Group=Test** | -0.001 | 0.0170 | -0.034 | 0.032 | 0.951 |
| **Group=Control (ref.)** | 0 | . | . | . | . |
| **Time** | -0.003 | 0.0008 | -0.004 | -0.001 | **0.001**** |
| **Group=Test*Time** | 0.000 | 0.0010 | -0.002 | 0.002 | 0.854 |
| **Group=Control*Time** | 0 | . | . | . | . |
| **(Scale)** | 0.004 |  |  |  |  |

*p<0.05; **p<0.01; ***p<0.001

Longitudinal regression analysis assessing **CDV FI_blue_ at the transverse aspect** using GEE model and the control group as reference category.

|  | **B** | **SE** | **95% Wald Confidence Interval** | |  |
| --- | --- | --- | --- | --- | --- |
|  |  |  | **Lower** | **Upper** | **p-value** |
| **Intercept** | 0.072 | 0.0066 | 0.060 | 0.085 | **<0.001***** |
| **Group=Test** | 0.015 | 0.0101 | -0.005 | 0.035 | 0.141 |
| **Group=Control (ref.)** | 0 | . | . | . | . |
| **Time** | -0.001 | 0.0006 | -0.002 | 0.000 | 0.103 |
| **Group=Test*Time** | -0.002 | 0.0008 | -0.003 | 0.000 | **0.021*** |
| **Group=Control*Time** | 0 | . | . | . | . |
| **(Scale)** | 0.003 |  |  |  |  |

*p<0.05; **p<0.01; ***p<0.001

Longitudinal regression analysis assessing **PDI FI at the transverse aspect** using GEE model and the control group as reference category.

|  | **B** | **SE** | **95% Wald Confidence Interval** | |  |
| --- | --- | --- | --- | --- | --- |
|  |  |  | **Lower** | **Upper** | **p-value** |
| **Intercept** | 0.578 | 0.0197 | 0.539 | 0.617 | <0.001*** |
| **Group=Test** | 0.029 | 0.0534 | -0.076 | 0.133 | 0.592 |
| **Group=Control (ref.)** | 0 | . | . | . | . |
| **Time** | -0.002 | 0.0034 | -0.009 | 0.005 | 0.571 |
| **Group=Test*Time** | -0.002 | 0.0044 | -0.011 | 0.007 | 0.654 |
| **Group=Control*Time** | 0 | . | . | . | . |
| **(Scale)** | 0.091 |  |  |  |  |

*p<0.05; **p<0.01; ***p<0.001

Longitudinal regression analysis assessing **ANG expression** using GEE model and the control group as reference category.

|  | **B** | **SE** | **95% Wald Confidence Interval** | |  |
| --- | --- | --- | --- | --- | --- |
|  |  |  | **Lower** | **Upper** | **p-value** |
| **Intercept** | 648.0 | 51.8 | 546.4 | 749.6 | **<0.001***** |
| **Group=Untreated** | -175.4 | 45.4 | -264.6 | -86.3 | **<0.001***** |
| **Group=Test** | -20.8 | 86.2 | -189.8 | 148.2 | 0.809 |
| **Group=Control (ref.)** | 0 | . | . | . | . |
| **Time** | -16.1 | 7.44 | -30.7 | -1.51 | **0.031*** |
| **Group=Untreated*Time** | 12.1 | 7.40 | -2.40 | 26.6 | 0.102 |
| **Group=Test*Time** | -8.2 | 10.6 | -29.0 | 12.5 | 0.437 |
| **Group=Control*Time** | 0 | . | . | . | . |
| **(Scale)** | 142950.7 |  |  |  |  |

*p<0.05; **p<0.01; ***p<0.001

Longitudinal regression analysis assessing **bFGF** using GEE model and the control group as reference category.

|  | **B** | **SE** | **95% Wald Confidence Interval** | |  |
| --- | --- | --- | --- | --- | --- |
|  |  |  | **Lower** | **Upper** | **p-value** |
| **Intercept** | 0.449 | 0.220 | 0.017 | 0.880 | 0.041 |
| **Group=Untreated** | 0.123 | 0.255 | -0.378 | 0.623 | 0.631 |
| **Group=Test** | 0.177 | 0.271 | -0.355 | 0.709 | 0.515 |
| **Group=Control (ref.)** | 0 | . | . | . | . |
| **Time** | 0.005 | 0.010 | -0.015 | 0.026 | 0.596 |
| **Group=Untreated*Time** | -0.007 | 0.017 | -0.041 | 0.026 | 0.670 |
| **Group=Test*Time** | -0.029 | 0.022 | -0.073 | 0.016 | 0.210 |
| **Group=Control*Time** | 0 | . | . | . | . |
| **(Scale)** | 1.967 |  |  |  |  |

*p<0.05; **p<0.01; ***p<0.001

Longitudinal regression analysis assessing **IL-1β** using GEE model and the control group as reference category.

|  | **B** | **SE** | **95% Wald Confidence Interval** | |  |
| --- | --- | --- | --- | --- | --- |
|  |  |  | **Lower** | **Upper** | **p-value** |
| **Intercept** | 9.18 | 1.94 | 5.37 | 12.9 | <0.001*** |
| **Group=Untreated** | -0.11 | 2.27 | -4.58 | 4.35 | 0.960 |
| **Group=Test** | 8.18 | 4.89 | -1.40 | 17.7 | 0.094 |
| **Group=Control (ref.)** | 0 | . | . | . | . |
| **Time** | -0.02 | 0.24 | -0.49 | 0.45 | 0.926 |
| **Group=Untreated*Time** | 0.15 | 0.29 | -0.42 | 0.73 | 0.606 |
| **Group=Test*Time** | -0.98 | 0.48 | -1.94 | -0.03 | **0.043*** |
| **Group=Control*Time** | 0 | . | . | . | . |
| **(Scale)** | 346.8 |  |  |  |  |

*p<0.05; **p<0.01; ***p<0.001

Longitudinal regression analysis assessing **IL-6** using GEE model and the control group as reference category.

|  | **B** | **SE** | **95% Wald Confidence Interval** | |  |
| --- | --- | --- | --- | --- | --- |
|  |  |  | **Lower** | **Upper** | **p-value** |
| **Intercept** | 49.7 | 20.2 | 10.0 | 89.5 | 0.014* |
| **Group=Untreated** | -48.0 | 20.3 | -87.9 | -8.18 | **0.018*** |
| **Group=Test** | 108.6 | 62.4 | -13.7 | 230.9 | **0.082*** |
| **Group=Control (ref.)** | 0 | . | . | . | **.** |
| **Time** | -4.65 | 1.91 | -8.40 | -0.89 | **0.015*** |
| **Group=Untreated*Time** | 4.69 | 1.92 | 0.93 | 8.46 | **0.014*** |
| **Group=Test*Time** | -10.3 | 5.92 | -21.9 | 1.25 | 0.081 |
| **Group=Control*Time** | 0 | . | . | . | . |
| **(Scale)** | 40304.5 |  |  |  |  |

*p<0.05; **p<0.01; ***p<0.001

Longitudinal regression analysis assessing **IL-10** using GEE model and the control group as reference category.

|  | **B** | **SE** | **95% Wald Confidence Interval** | |  |
| --- | --- | --- | --- | --- | --- |
|  |  |  | **Lower** | **Upper** | **p-value** |
| **Intercept** | 0.20 | 0.03 | 0.13 | 0.26 | <0.001*** |
| **Group=Untreated** | -0.08 | 0.04 | -0.16 | 0.00 | 0.054 |
| **Group=Test** | 0.25 | 0.18 | -0.10 | 0.61 | 0.163 |
| **Group=Control (ref.)** | 0 | . | . | . | . |
| **Time** | -0.00 | 0.00 | -0.01 | 0.00 | 0.352 |
| **Group=Untreated*Time** | 0.01 | 0.01 | -0.01 | 0.04 | 0.214 |
| **Group=Test*Time** | -0.01 | 0.01 | -0.04 | 0.01 | 0.341 |
| **Group=Control*Time** | 0 | . | . | . | . |
| **(Scale)** | 0.35 |  |  |  |  |

*p<0.05; **p<0.01; ***p<0.001

Longitudinal regression analysis assessing **PDGF-BB** using GEE model and the control group as reference category.

|  | **B** | **SE** | **95% Wald Confidence Interval** | |  |
| --- | --- | --- | --- | --- | --- |
|  |  |  | **Lower** | **Upper** | **p-value** |
| **Intercept** | 0.61 | 0.17 | 0.27 | 0.96 | <0.001*** |
| **Group=Untreated** | 0.46 | 0.25 | -0.03 | 0.95 | 0.067 |
| **Group=Test** | 4.90 | 1.63 | 1.69 | 8.11 | **0.003**** |
| **Group=Control (ref.)** | 0 | . | . | . | . |
| **Time** | -0.01 | 0.02 | -0.05 | 0.03 | 0.616 |
| **Group=Untreated*Time** | 0.02 | 0.04 | -0.05 | 0.10 | 0.522 |
| **Group=Test*Time** | -0.44 | 0.15 | -0.74 | -0.14 | **0.003**** |
| **Group=Control*Time** | 0 | . | . | . | . |
| **(Scale)** | 30.9 |  |  |  |  |

*p<0.05; **p<0.01; ***p<0.001

Longitudinal regression analysis assessing **TGF β1** using GEE model and the control group as reference category.

|  | **B** | **SE** | **95% Wald Confidence Interval** | |  |
| --- | --- | --- | --- | --- | --- |
|  |  |  | **Lower** | **Upper** | **p-value** |
| **Intercept** | 7056.1 | 3063.9 | 1050.9 | 13061.4 | 0.021* |
| **Group=Untreated** | -2197.3 | 1717.3 | -5563.3 | 1168.6 | 0.201 |
| **Group=Test** | -529.3 | 5408.8 | -11130.5 | 10071.7 | 0.922 |
| **Group=Control (ref.)** | 0 | . | . | . | . |
| **Time** | -399.0 | 267.7 | -923.8 | 125.6 | 0.136 |
| **Group=Untreated*Time** | 476.0 | 354.9 | -219.6 | 1171.7 | 0.180 |
| **Group=Test*Time** | -66.8 | 505.6 | -1057.8 | 924.2 | 0.895 |
| **Group=Control*Time** | 0 | . | . | . | . |
| **(Scale)** | 25147037 |  |  |  |  |

*p<0.05; **p<0.01; ***p<0.001

Longitudinal regression analysis assessing **TIMP-2** using GEE model and the control group as reference category.

|  | **B** | **SE** | **95% Wald Confidence Interval** | |  |
| --- | --- | --- | --- | --- | --- |
|  |  |  | **Lower** | **Upper** | **p-value** |
| **Intercept** | 835,9 | 98,9 | 642,1 | 1029,8 | <0,001*** |
| **Group=Untreated** | -59,9 | 83,7 | -224,0 | 104,2 | 0,474 |
| **Group=Test** | -29,2 | 132,9 | -289,8 | 231,2 | 0,826 |
| **Group=Control (ref.)** | 0 | . | . | . | . |
| **Time** | -20,4 | 8,55 | -37,2 | -3,65 | **0,017*** |
| **Group=Untreated*Time** | 12,8 | 10,5 | -7,77 | 33,5 | 0,222 |
| **Group=Test*Time** | 1,41 | 13,3 | -24,6 | 27,4 | 0,916 |
| **Group=Control*Time** | 0 | . | . | . | . |
| **(Scale)** | 207277,9 |  |  |  |  |

*p<0.05; **p<0.01; ***p<0.001

Longitudinal regression analysis assessing **TNF-α** using GEE model and the control group as reference category.

|  | **B** | **SE** | **95% Wald Confidence Interval** | |  |
| --- | --- | --- | --- | --- | --- |
|  |  |  | **Lower** | **Upper** | **p-value** |
| **Intercept** | 747.6 | 173.9 | 406.6 | 1088.5 | <0.001*** |
| **Group=Untreated** | -276.8 | 125.3 | -522.5 | 31.1 | 0.110 |
| **Group=Test** | 125.1 | 429.7 | -717.1 | 967.4 | 0.771 |
| **Group=Control (ref.)** | 0 | . | . | . | . |
| **Time** | -13.5 | 20.6 | -53.9 | 26.9 | 0.513 |
| **Group=Untreated*Time** | 46.9 | 34.6 | -20.9 | 114.9 | 0.175 |
| **Group=Test*Time** | -34.7 | 40.0 | -113.3 | 43.7 | 0.386 |
| **Group=Control*Time** | 0 | . | . | . | . |
| **(Scale)** | 1755879 |  |  |  |  |

*p<0.05; **p<0.01; ***p<0.001

Longitudinal regression analysis assessing **VEGF** using GEE model and the control group as reference category.

|  | **B** | **SE** | **95% Wald Confidence Interval** | |  |
| --- | --- | --- | --- | --- | --- |
|  |  |  | **Lower** | **Upper** | **p-value** |
| **Intercept** | 40.6 | 7.06 | 26.7 | 54.4 | <0.001*** |
| **Group=Untreated** | -4.39 | 8.36 | -20.7 | 12.0 | 0.600 |
| **Group=Test** | 34.1 | 16.8 | 1.22 | 67.1 | **0.042*** |
| **Group=Control (ref.)** | 0 | . | . | . | . |
| **Time** | -1.00 | 0.69 | -2.36 | 0.35 | 0.147 |
| **Group=Untreated*Time** | 0.67 | 0.82 | -0.94 | 2.30 | 0.413 |
| **Group=Test*Time** | -4.04 | 1.57 | -7.12 | -0.95 | **0.010*** |
| **Group=Control*Time** | 0 | . | . | . | . |
| **(Scale)** | 3501.6 |  |  |  |  |

*p<0.05; **p<0.01; ***p<0.001
